# Acetylation of AGO2 promotes cancer progression by increasing oncogenic miR-19b biogenesis

**DOI:** 10.1038/s41388-018-0530-7

**Published:** 2018-10-10

**Authors:** Hailong Zhang, Yanli Wang, Jinzhuo Dou, Yanmin Guo, Jianfeng He, Lian Li, Xiaojia Liu, Ran Chen, Rong Deng, Jian Huang, Ruiyu Xie, Xian Zhao, Jianxiu Yu

**Affiliations:** 1 Department of Biochemistry and Molecular Cell Biology, Shanghai Key Laboratory of Tumor Microenvironment and Inflammation, Shanghai Jiao Tong University School of Medicine, Shanghai, 200025 China; 2 State Key Laboratory of Oncogenes and Related Genes, Shanghai Jiao Tong University School of Medicine, Shanghai, 200025 China; 3 Department of Pathophysiology, Key Laboratory of Cell Differentiation and Apoptosis of Chinese Ministry of Education, Shanghai Jiao Tong University School of Medicine, Shanghai, 200025 China; 4 Faculty of Health of Sciences, University of Macau, Macau, 999078 China

**Keywords:** Non-coding RNAs, Acetylation

## Abstract

Argonaute2 (AGO2) is an effector of small RNA mediated gene silencing. Increasing evidence show that post-translational modifications of AGO2 can change miRNA activity at specific or global levels. Among the six mature miRNAs that are encoded by miR-17-92, miR-19b1 is the most powerful to exert the oncogenic properties of the entire cluster. Here we identify that AGO2 can be acetylated by P300/CBP and deacetylated by HDAC7, and that acetylation occurs at three sites K720, K493, and K355. Mutation of K493R/K720R, but not K355R at AGO2, inhibits miR-19b biogenesis. We demonstrate that acetylation of AGO2 specifically increases its recruiting pre-miR-19b1 to form the miPDC (miRNA precursor deposit complex), thereby to enhance miR-19b maturation. The motif UGUGUG in the terminal-loop of pre-miR-19b1, as a specific processing feature that is recognized and bound by acetylated AGO2, is essential for the assembly of miRISC (miRNA-induced silencing complex) loading complex. Analyses on public clinical data, xenograft mouse models, and IHC and ISH staining of lung cancer tissues, further confirm that the high levels of both AGO2 acetylation and miR-19b correlate with poor prognosis in lung cancer patients. Our finding reveals a novel function of AGO2 acetylation in increasing oncogenic miR-19b biogenesis and suggests that modulation of AGO2 acetylation has potential clinical implications.

## Introduction

MicroRNAs (miRNAs) have crucial roles in cancer initiation, progression, and metastasis [1]. The miR-17-92 cluster, comprised by miR-17, miR-18a, miR-19a, miR-20a, miR-19b1, and miR-92a1, is well known as key oncogenes in promoting tumorigenesis and cancer cell proliferation [2]. Notably, ectopical expression of the miR-17-92 cluster accelerates tumor development in a mouse B-cell lymphoma model, which is firstly distinguished as ‘*oncomiR*’ [3]. The miR-17-92 cluster is also highly expressed in embryonic cells and the deletion of the miR-17-92 cluster is perinatal lethal from cardiac defects and lung hypoplasia [4, 5]. Among the six mature miRNAs that are encoded by miR-17-92, miR-19b is the most powerful to exert the oncogenic properties of the entire cluster [6, 7] by repressing expression of tumor suppressers such as PTEN [7, 8] and P53 [9]. However, it is quite unclear how miR-19b biogenesis is regulated in tumor cells.

The microRNA (miRNA) expression levels are globally suppressed in human cancer compared to those in normal tissues [10], which are mostly contributed to genetic and epigenetic alterations in miRNA-processing machinery components such as DROSHA, DGCR8, XPO5, TARBP2, DICER, and AGO2 in human cancer [1]. A good example is that the phosphorylation of exportin-5 (XPO5) by high-activated ERK can inhibit the recruiting and nuclear-export of pre-miRNA, and thereby globally downregulates miRNA processing in hepatocellular carcinoma (HCC) [11].

Among the four mammalian Argonaute proteins, AGO2 is the unique one endowed with nuclease activity, which is essential for cleavage of not only the passenger strand of miRNA/siRNA (small interfering RNA) duplexes but also RNA targeted by siRNA/miRNA [12, 13]. The ribonuclease activity of AGO2 is also critical for direct cleavage of the 3’-arm of pre-miR-451 into mature miR-451, which is an alternative DICER-independent miRNA processing way [14–16]. Moreover, in addition to DICER cleavage of pre-miRNAs, AGO2 can also cleave the hairpin 12 nucleotides from the 3’-end of a subset of pre-miRNAs to the additional processing intermediates, termed as AGO2-cleaved precursor miRNAs (ac-pre-miRNAs), which subsequently serve as substrates for DICER to mature into the active miRNAs [17]. The canonical miRLC formation is thought to be the pre-miRNA binding to a performed complex of DICER, TARBP2, and AGO2. Interestingly, AGO2 can directly interact with pre-miRNAs in vivo and in vitro to form Ago2:pre-miRNA complexes even in the absence of DICER [18]. It has also been proposed that certain miRLCs might be preceded by formation of miRNA precursor deposit complex (miPDC), in which AGO2 directly interacts with and recruits pre-miRNAs [19]. The miPDC formation plays a critical role in DICER-independent miRNA (such as miR-451) biogenesis and promoting miRLC assembly of certain DICER-dependent miRNAs, which is necessary for further miRNA processing from pre-miRNAs to mature miRNAs [19]. Yet, the mechanistic details of AGO2 recruiting these pre-miRNAs for miPDC formation and afterwards maturation is still unclear.

AGO2 can undergo a variety of post-translational modifications (PTMs), including prolyl-4-hydroxylation [20, 21], phosphorylation [22–26], ubiquitination [27], PARylation (poly-ADP-ribosylation) [28], and SUMOylation [29]. PTMs of AGO2 play key roles in either miRNA biogenesis or miRNA-guided gene silencing at the global or specific levels. For examples, prolyl-4-hydroxylation at P700 as the firstly identified PTM of AGO2 is important for AGO2 stability and effective RISC activity [20, 21]. More interestingly, phosphorylation of AGO2 at Y393 mediated by EGFR reduces the binding of DICER to AGO2 in turn to inhibit miRNA processing from pre-miRNAs to mature miRNAs [23]. Phosphorylation of AGO2 at S824-S834 by CSNK1A1 impairs miRNA-mediated gene silencing through inhibition its interaction with target mRNA [24, 26]. AGO2 Ubiquitination mediated by an E3 ubiquitin ligase mLin41 regulates AGO2-miRISC turnover [27]. In addition, PARylation of AGO2 relieves both miRNA-mediated translational repression and miRNA-directed mRNA cleavage [28]. Collectively, AGO2 acts as an efficient modulator in regulating miRNA processing, miRNA-guided gene silencing and other functions via its different PTMs. In this study, we identify a novel PTM of AGO2, acetylation, and demonstrate that acetylation of AGO2 promotes cancer progression by specifically increasing miR-19b biogenesis.

## Results

### AGO2 is acetylated at K355, K493, and K720

**Fig. 1:**
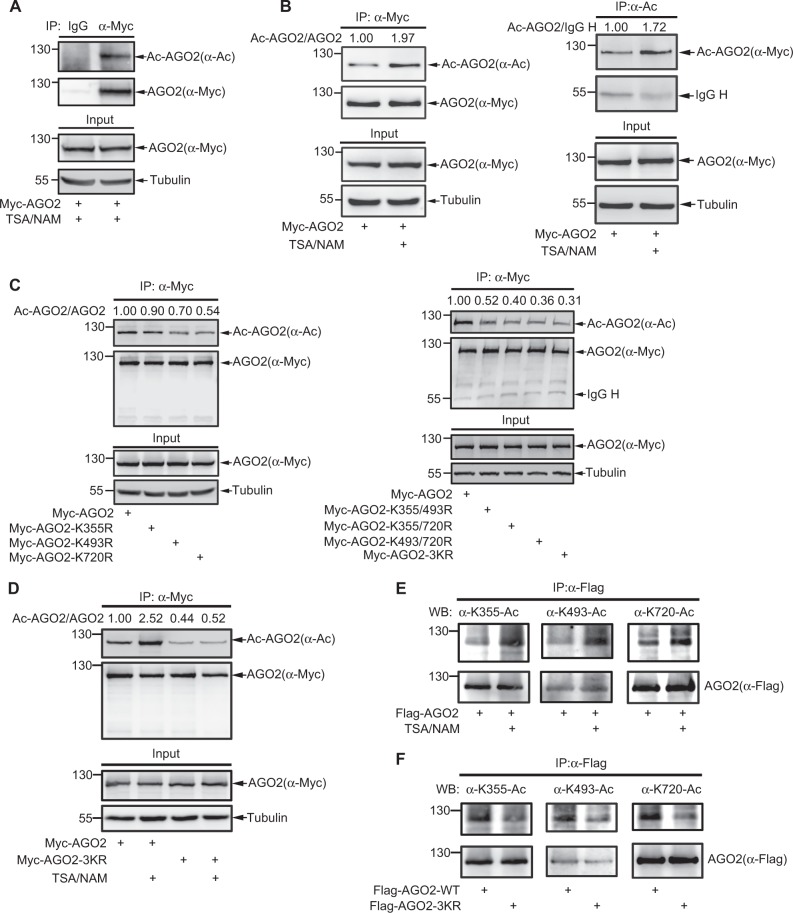
AGO2 Is Acetylated at K355, K493 and K720. **a**, **b** AGO2 is acetylated. 293T cells transfected with Myc-AGO2 were treated with the deacetylase inhibitors TSA (2 μM) and NAM (10 mM) for 6 h and 18 h before harvested, respectively. Cell lysates were used for IP with anti-Myc antibody or anti-acetylated-Lysine (anti-Ac) or normal mouse IgG (as a negative control), and then AGO2 acetylation was analyzed by Western blotting (WB) with as indicated antibodies. **c** Single, double and triple mutations of K355, K493 and K720 reduce AGO2 acetylation. The indicated single (left panels), double and triple (right panels) mutations of Myc-AGO2 were transfected into 293T cells. AGO2 acetylation was detected by IP/WB. **d** TSA/NAM increases the acetylation level of AGO2-WT but not of AGO2-3KR. 293T cells transfected with AGO2-WT or AGO2-3KR were treated with or without TSA/NAM as (**a**). AGO2 acetylation was analyzed by IP/WB. **e** TSA/NAM increases AGO2 acetylations at K355, K493 and K720. 293T cells transfected with Flag-AGO2 were treated with or without TSA/NAM as in (**a**). Cell lysates were IP with anti-Flag antibody, followed by WB with home-made AGO2 specific acetyl-antibodies. **f** Triple-mutation of AGO2 at K355, K493 and K720 impairs its acetylation. AGO2-WT and AGO2-3KR were transfected into 293T cell, and then AGO2 acetylation was analyzed by IP/ WB with AGO2 specific acetyl-antibodies. See Figure S1A for three acetylation sites K355, K493 and K720 identified by mass spectrometry, Figure S1B-D for the specificity of home-made AGO2 specific acetylation antibodies AGO2-K355-Ac, AGO2-K493-Ac and AGO2-K720-Ac

To determine whether AGO2 is acetylated, we transfected Myc-tagged AGO2 into human embryonic kidney (HEK) 293T cells and then treated with trichostatin A (TSA) and nicotinamide (NAM), which are respectively the histone deacetylase (HDAC) family I /II/IV inhibitor and the SIRT family deacetylase inhibitor. Immunoprecipitates with anti-Myc antibody following by immunoblotting with anti-acetylated lysine (anti-Ac) showed that AGO2 was acetylated (Fig. 1a) and the levels of acetylated AGO2 was increased after the treatment with TSA/NAM (Fig. 1b, left panels). Reciprocally, immunoprecipitates with anti-Ac antibody were detected with anti-Myc, also revealing that AGO2 acetylation was enhanced by TSA/NAM treatment (Fig. 1b, right panels). These results suggest that AGO2 is acetylated.

To identify the acetylation sites of AGO2, purified ectopically expressed Flag-tagged AGO2 by immunoprecipitation was analyzed by using mass spectrometry. The potential acetylation sites of AGO2 were identified at the lysine (K)-355, -493, and -720 (Figure S1A). The alignment analysis of amino acid sequences in AGO2 homologs from different species showed that K355, K493, and K720 are evolutionarily conserved from *Caenorhabditis elegans* to mammals (Table S1). Next we attempted to verify these three acetylation sites of AGO2 by point-mutations. Single mutation of each lysine to arginine (R) resulted in a weak attenuation in AGO2 acetylation, while combination of double mutations attenuated more and triple mutations of all this three residues (AGO2-3KR) resulted in a large decrease in AGO2 acetylation (Fig. 1c). Moreover, the acetylation level of the wild-type AGO2 but not of AGO2-3KR was greatly increased with the treatment of TSA/NAM (Fig. 1d). All these results demonstrate that AGO2 is majorly acetylated at K355, K493, and K720.

To further confirm acetylation of the three residues, we generated antibodies specifically against acetylated K355, K493, and K720. To characterize the specificity of these three antibodies, we performed the dot-blot assays and found that anti-AGO2 acetyl-K355,-K493, and -K720 antibodies preferentially detected the acetylated peptide, but not the unmodified peptide, respectively (Figure S1B). Further we confirmed that all of these three specific acetyl-antibodies did not across with each other (Figure S1C). The immunoprecipitated complexes of ectopically expressed Flag-AGO2 with anti-Flag antibody were easily detected by these AGO2 specific acetyl-antibodies K355-Ac, K493-Ac, and K720-Ac, which were competitively weakened by addition of the corresponding specific acetyl-modified peptides, respectively (Figure S1D). Moreover, by using these home-made AGO2 acetyl-antibodies, we found that the acetylation levels of AGO2 at K355, K493, and K720 were significantly increased in 293T cells after the treatment with TSA/NAM (Fig. 1e). In addition, immunoprecipitates of ectopically expressed Flag-AGO2-WT, but not the mutant AGO2-3KR, were be strongly detected by these antibodies (Fig. 1f). Taken together, these results confirm that AGO2 is acetylated at three major sites K355, K493, and K720.

### P300/CBP acetylate AGO2

**Fig. 2:**
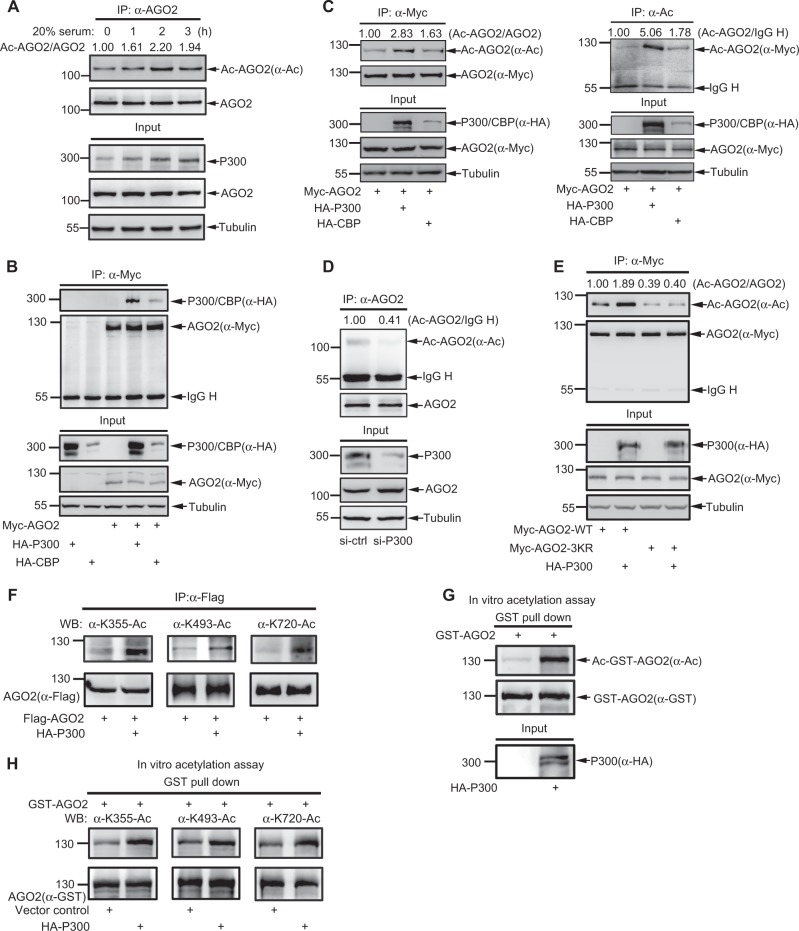
P300/CBP acetylate AGO2. **a** Serum stimulates AGO2 acetylation. 293T cells were serum-starved for 24 h and followed by stimulation with 20% serum for 1, 2, 3 h; AGO2 acetylation was measured by IP/WB. **b** P300 and CBP interact with AGO2. HA-P300 or HA-CBP was co-transfected with or without Myc-AGO2 into 293T cells. The association between AGO2 and P300 or CBP was determined by IP/WB. Also see Figure S2B. **c** Overexpression of P300/CBP significantly increases AGO2 acetylation. Myc-AGO2 with HA-P300 or HA-CBP were transfected into 293T cells. AGO2 acetylation was determined by IP with anti-Myc antibody (left panel) or anti-Ac antibody (right panel), and followed by WB. **d** Knockdown of P300 attenuates AGO2 acetylation. P300-siRNA (40 nM) was transfected into 293T cells for 48 h, and AGO2 acetylation were examined by IP/WB. **e** P300 significantly increases acetylation of AGO2-WT rather than AGO2-3KR. Myc-tagged AGO2-WT or AGO2-3KR was co-transfected with or without HA-P300 into 293T cells, and then AGO2 acetylation was analyzed by IP with anti-Myc antibody and WB with anti-Ac antibody. **f** P300 mediates AGO2 acetylation at K355, K493 and K720. Flag-tagged AGO2 was co-transfected with or without HA-P300 into 293T cells, and AGO2 acetylation was analyzed by IP with anti-Flag antibody and WB with AGO2 specific acetyl-antibodies. **g**, **h** P300 increases AGO2 acetylation in vitro. Purified GST-AGO2 protein was incubated in cell lysates of 293T cells expressing HA-P300 or the control vector. Acetylation of GST-AGO2 was analyzed by GST pull down and followed by WB with anti-Ac antibody (**g**) or AGO2 specific acetyl-antibodies (**h**)

To determine whether AGO2 acetylation can be induced in cells, 293T cells were deprived of serum for 24 h and then re-added 20% serum for indicated times. We found that AGO2 acetylation was significantly increased under serum stimulation in a time-course manner (Fig. 2a). P300 (an E1A-binding protein) induction was used as a positive control (Fig. 2a), since P300 and CBP (a cAMP response element-binding protein) can be induced through a transcription factor EGR1 under serum stimulation, as previously reported by us [30]. We further confirmed the expression of P300 was also induced in other two cell lines HeLa and A549 by serum stimulation (Figure S2A). We noticed that the induction of AGO2 acetylation was very consistent with the expression level of P300 induced by serum, indicating that P300 is a potential acetyltransferase for AGO2. To confirm this, we co-transfected Myc-tagged AGO2 with P300 or CBP into 293T cells, and found that both ectopically expressed P300 and CBP associated with exogenous AGO2 (Fig. 2b) through the method of co-immunoprecipitation (co-IP) with anti-Myc antibody. In addition, we also observed that the ectopically expressed HA-tagged P300 also associated with endogenous AGO2 (Figure S2B). Thus, these results imply that P300/CBP are acetyltransferases for AGO2.

To further investigate whether P300/CBP influence AGO2 acetylation, 293T cells transfected with Myc-tagged AGO2 with either P300 or CBP were lysed for co-IP with either anti-Myc (Fig. 2c, left panels) or anti-Ac (Fig. 2c, right panels), and followed by immunoblotting analysis. The results showed overexpression of P300 increased AGO2 acetylation, whereas moderate-expression of CBP had still effect on it (Fig. 2c). Reversely, we knocked down *p300* by siRNA and found that endogenous AGO2 acetylation was obviously reduced (Fig. 2d).

To verify whether P300 acetylates AGO2 at K355, K493, and K720, we transfected AGO2-WT and AGO2-3KR with or without P300 into 293T cells, and found that co-expression of P300 increased the acetylation of AGO2-WT approximately twofold, but it did not affect AGO2-3KR mutant acetylation (Fig. 2e). Consistent with this, P300 greatly increased AGO2 acetylations at K355, K493, and K720 by IP/WB detection with AGO2 specific acetyl-antibodies K355-Ac, K493-Ac, and K720-Ac (Fig. 2f). Moreover, the in vitro acetylation assay showed that bacterially expressed glutathione S-transferase (GST)-tagged AGO2 fusion protein was acetylated much higher in incubation with lysates from 293T cells overexpressing P300 than that from cells transfected the control vector (Fig. 2g). The acetylation levels at all three sites K355, K493, and K720 of GST-AGO2 were obviously increased in incubation with lysates from cells overexpressing P300 compared to that from cells transfected with the control vector (Fig. 2h). Therefore, our results validate that P300 is an acetyltransferase for AGO2.

### HDAC7 deacetylates AGO2

**Fig. 3:**
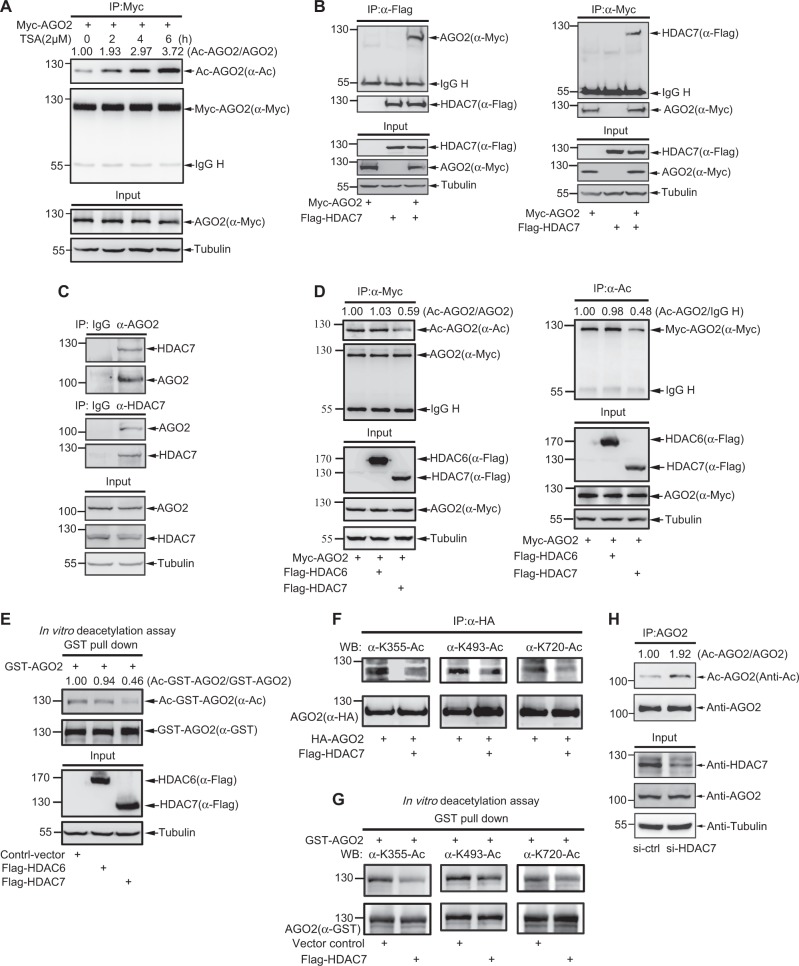
HDAC7 Deacetylates AGO2. **a** An HDAC family specific inhibitor TSA increases AGO2 acetylation. 293T cells transfected Myc-AGO2 were treated with 2 μM TSA for indicated time before harvested. AGO2 acetylation was determined by IP/WB. **b** The association between exogenous AGO2 and HDAC7. Myc-AGO2 and Flag-HDAC7 were co-transfected into 293T cells, and the association of AGO2 with HDAC7 was determined by IP with anti-Flag (left panels) and anti-Myc (right panels) antibodies, and followed by WB. Also see Figure S3. **c** The association between endogenous AGO2 and HDAC7. Lysates from 293T cells were immunoprecipitated with anti-AGO2 (upper panels) and anti-HDAC7 antibodies (middle panels) (normal IgG as a negative control), then followed by WB. **d** HDAC7 rather than HDAC6 decreases AGO2 acetylation. Myc-AGO2 was co-transfected with Flag-HDAC6 or Flag-HDAC7 into 293T cells. The acetylation levels of AGO2 were analyzed by IP/WB. **e** HDAC7 but not HDAC6 decreases AGO2 acetylation in vitro. Purified GST-AGO2 protein was incubated in lysates from 293T cells expressing Flag-HDAC6, Flag-HDAC7 or the control vector. Acetylation of GST-AGO2 was analyzed by GST pull down and followed by WB. **f** HDAC7 decreases AGO2 acetylation at K355, K493 and K720. HA-tagged AGO2 was co-transfected with or without Flag-HDAC7 into 293T cells, and AGO2 acetylation was analyzed by IP and WB with AGO2 specific acetyl- antibodies. **g** HDAC7 decreases AGO2 acetylation at K355, K493 and K720 in vitro. Purified GST-AGO2 protein was incubated in cell lysates of 293T cells expressing Flag-HDAC7 or the control vector. Acetylation of GST-AGO2 was analyzed by GST pull down and following WB with AGO2 specific acetyl-antibodies. **h** Knockdown of HDAC7 increases AGO2 acetylation. HDAC7-siRNA (40 nM) was transfected into 293T cells for 48 h, and AGO2 acetylation were examined by IP/WB

To identify the deacetylase of AGO2, we treated 293T cells overexpressing Myc-AGO2 with the inhibitor TSA and found that the level of AGO2 acetylation was increased in a time-dependent manner (Fig. 3a), suggesting that HDAC family members are involved in AGO2 deacetylation. Then we co-transfected Myc-AGO2 with SIRT1, SIRT5, HDAC6 or HDAC7, into 293T cells, and found that HDAC7 associated with AGO2 (Figure S3). To further confirm this, lysates from 293T cells transfected with Myc-AGO2, Flag-HDAC7 or both were used for co-IP with anti-Flag or anti-Myc antibody, respectively. The following immunoblotting results showed that AGO2 indeed interacted with HDAC7 (Fig. 3b). More convincingly, the interaction between endogenous AGO2 and HDAC7 in 293T cells were detected by co-IP with either anti-AGO2 or anti-HDAC7 antibody (Fig. 3c). These results suggest that HDAC7 is a potential deacetylase of AGO2.@@@

To test whether HDAC7 mediates de-acetylation of AGO2, we co-expressed Myc-AGO2 with either Flag-HDAC7 or Flag-HDAC6 into 293T cells. Through the similar reciprocal co-IP with anti-Myc or anti-Ac antibody and immunoblotting, as expectedly, we found that AGO2 acetylation was suppressed by HDAC7 but not by HDAC6 (Fig. 3d). Significantly, the in vitro deacetylation assays revealed that the acetylation level of GST-AGO2 in incubation with lysates from HDAC7-transfected 293T cells was greatly attenuated compared to those in incubation with lysates from the control vector- or HDAC6- transfected (Fig. 3e). Moreover, by using the three AGO2 specific acetyl-antibodies we found that overexpression of HDAC7 dramatically decreased AGO2 acetylation at three sites K355, K493 and K720 (Fig. 3f). Being in line with this, the in vitro deacetylation assays showed that the acetylation levels at K355, K493 and K720 of GST-AGO2 were greatly decreased by incubation in lysates from cells expressing HDAC7 compared to those from cells transfected with the control vector (Fig. 3g). Additionally, we knocked down HDAC7 by using siRNA and found that endogenous AGO2 acetylation was obviously increased (Fig. 3h). Therefore, these results demonstrate that HDAC7 is responsible for the deacetylation of AGO2.

### Acetylation of AGO2 promotes miR-19b biogenesis

**Fig. 4:**
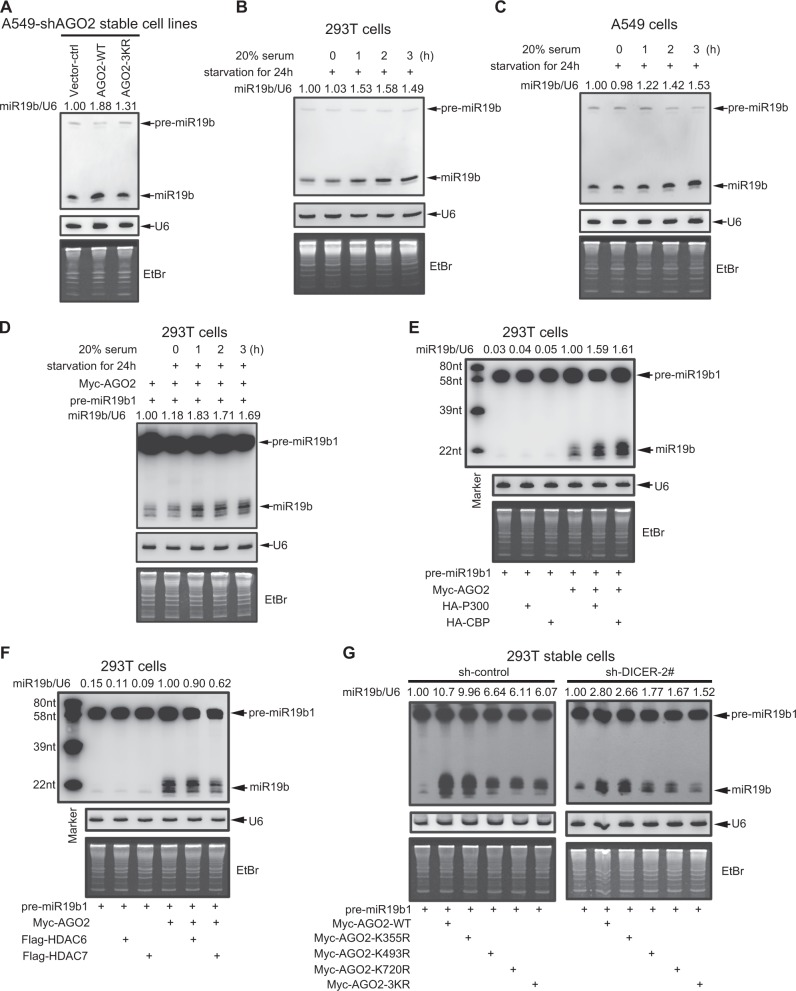
Acetylation of AGO2 increases miR-19b biogenesis. **a** The expression of miR-19b in A549-shAGO2 cells re-expressing Flag-AGO2-WT was much more than that in cells re-expressing Flag-AGO2-3KR. Total RNA was extracted from A549-stable cell lines, the miR-19b expression were detected by Northern blotting. See Figure S4A for the expression levels of AGO2-WT, AGO2-3KR were comparable. Also see Supplementary Data S1 for the results of small RNA high-throughput deep sequencing. **b**–**d** Serum stimulates miR-19b biogenesis. 293T cells (**b**), A549 cells (**c**) or 293T cells overexpressing indicated plasmids (**d**) were serum-starvated for 24 h and then stimulated by 20% serum as indicated times. The total RNA were extracted and followed by northern blotting analysis. See Figure S5 for the expression levels of pri-miR-19b1, pre-miR-19b1 and miR-19b by QRT-PCR analysis. **e**, **f** P300/CBP increases miR-19b maturation from pre-miR-19b1 mediated by AGO2 while HDAC7 decreases it. 293T cells were transfected with indicated plasmids, 48 h later the total RNA were extracted and followed by northern blotting analysis. **g** Acetyl-mutants K493R and K720R, but not K355R of AGO2 decrease miR-19 biogenesis in a DICER dependent manner. DICER-knocked down 293T stable cells were transfected with indicated plasmids, 48 h later, the total RNA were extracted and followed by northern blotting analysis. See Figure S4B–D for miR-19b maturation from pre-miR-19b1 is highly sensitive to AGO2 in a DICER dependent manner

To identify whether AGO2 acetylation takes participate in miRNA biogenesis, we stably re-expressed Flag-tagged AGO2-WT, AGO2-3KR and the control vector in an AGO2-knocked down A549 cell line (A549-shAGO2) by the lentiviral system (Figure S4A). Total RNAs were extracted from these stable A549 cell lines for high-throughput deep sequencing of small RNAs, and consequently a total of 1086 miRNAs were identified. In view of the expression levels with TPM (transcript per million), there were 248 miRNAs with TPM in AGO2-WT > 10 for follow-up analysis (Supplementary Data S1A). Then, we defined a 1.5-fold change of the miRNA expression level as the classification criterion. Compared with the control vector, 67 miRNAs were increased in AGO2-WT re-expression in A549-shAGO2 cells (AGO2-WT/Ctrl-Vector > 1.5 fold), and their expression levels were considered to be regulated by AGO2 (Supplementary Data S1B). Furthermore, the expression of six miRNAs including miR-19b-3p, miR-19a-3p, miR-339-5p, miR-18a-5p, miR-590-3p and miR-29a-5p (AGO2-WT/Ctrl-Vector > 1.5, AGO2-WT/AGO2-3KR > 1.5 fold) in A549-shAGO2 re-expressed AGO2-3KR cells were significantly decreased when compared to those of in A549-shAGO2 re-expressed AGO2-WT cells (Supplementary Data S1C). According to TPM of miRNAs, miR-19b-3p (miR-19b) was the most abundant among them (Supplementary Data S1D), indicating that AGO2 acetylation could be potentially involved in the mature processes of miR-19b. By performing the northern blotting, we confirmed the pattern of miR-19b expression levels (Fig. 4a) in above A549 stable cells was similar to the result from the high-throughput deep sequencing.

MiR-19b can be processed from completely different two precursors, pre-miR-19b1 and pre-miR-19b2, whose genes are located on chromosome 13 (MI0000074) and on chromosome X (MI000075), respectively, so we tried to figure out whether AGO2 has an influence on miR-19b maturation from one or both precursors. We transfected pre-miR-19b1 or pre-miR-19b2 into DICER- or AGO2-knocked down 293 T cells (Figure S4B) and found that knockdown of either DICER or AGO2 impaired miR-19b maturation from both pre-miR-19b1 and pre-miR-19b2 to varying degrees (Figure S4C). However, when we co-transfected pre-miR-19b1 or pre-miR-19b2 with AGO2 or DICER into 293 T cells to determine the yield of mature miR-19b, showing that overexpression of AGO2 enormously enhanced miR-19b maturation from either pre-miR-19b1 or pre-miR-19b2, while overexpression of DICER almost did not affect, indicating that miR-19b processing is specifically and highly sensitive to AGO2. But most importantly, AGO2-mediated miR-19b processing from pre-miR-19b1 was much stronger than that of from pre-miR-19b2 (Figure S4D), suggesting that most of miR-19b processing by AGO2 might come from pre-miR-19b1. Taken together, above results demonstrate that miR-19b maturation from pre-miR-19b1 rather than from pre-miR-19b2, is highly sensitive to AGO2 in a DICER dependent manner.

Since AGO2 acetylation was stimulated by serum (Fig. 2a), we wanted to test whether endogenous mature miR-19b is also increased. Under the same condition as in Fig. 2a, indeed we found that endogenous miR-19b biogenesis but not pre-miR-19b was increased in both 293 T (Fig. 4b) and A549 (Fig. 4c) cells by serum stimulation in a time dependent manner, suggesting that AGO2 acetylation increases miR-19b production. To further explore whether pri-miR-19b1/pre-miR-19b1 was induced in A549 cells by serum stimulation, qRT-PCR were performed to show that only mature miR-19b but not pri-miR-19b1 and pre-miR-19b1 was induced (Figure S5), indicating that AGO2 acetylation increases miR-19b maturation from pre-miR-19b1. The similar experiments with co-transfection of pre-miR-19b1 and AGO2 into 293 T cells were performed to show that miR-19b production from pre-miR-19b1 was significantly enhanced by serum stimulation (Fig. 4d). Moreover, total RNAs extracted from 293 T cells co-transfected with pre-miR-19b1 and AGO2 with/without CBP, P300, HDAC6 or HDAC7 were determined by northern blotting (Fig. 4e, f) to show that AGO2 acetylation mediated by CBP/P300 (see Fig. 2) significantly increased while AGO2 deacetylation by HDAC7 but not HDAC6 (see Fig. 3) obviously decreased the mature miR-19b biogenesis. Taken together, all above results reveal that AGO2 acetylation can promote miR-19b biogenesis.

To further investigate which acetyl-sites of AGO2 have an effect on miR-19b maturation from pre-miR-19b1, we co-transfected pre-miR-19b1 with AGO2-WT, AGO2-K355R, AGO2-K493R, AGO2-K720R or AGO2-3KR into 293T-shControl or 293T-shDICER stable cell lines, respectively. Northern blotting analysis showed that acetyl-mutants AGO2-K493R, AGO2-K720R and AGO2-3KR, but not AGO2-K355R, greatly reduced the production of mature miR-19b compared to that of AGO2-WT in both cell lines, although on the whole biogenesis of mature miR-19b in 293T-shDICER cells was much lesser than that in 293T-shControl cells (Fig. 4g). These results reveal that the acetyl-site mutations of K493R and K720R of AGO2 impair its mediating miR-19b biogenesis, in which DICER is required for pre-miR-19b1 processing to mature miR-19b.

### Acetelation of AGO2 enhances its recruiting pre-miR-19b1

We wanted to explore the underlying mechanism of AGO2 acetylation regulating miR-19b biogenesis. To test whether AGO2 acetylation influence its interaction with DICER, we transfected Myc-AGO2-WT, -K355R, -K493R, -K720R or -3KR individually into 293 T cells, and then performed co-immunoprecipitation with Myc-antibody, the interaction of endogenous of DICER with AGO2 were immunoblotted with DICER antibody. The result showed that acetylation at K355, K493 and K720 did not influence the interaction of AGO2 with DICER (Figure S6).

**Fig. 5:**
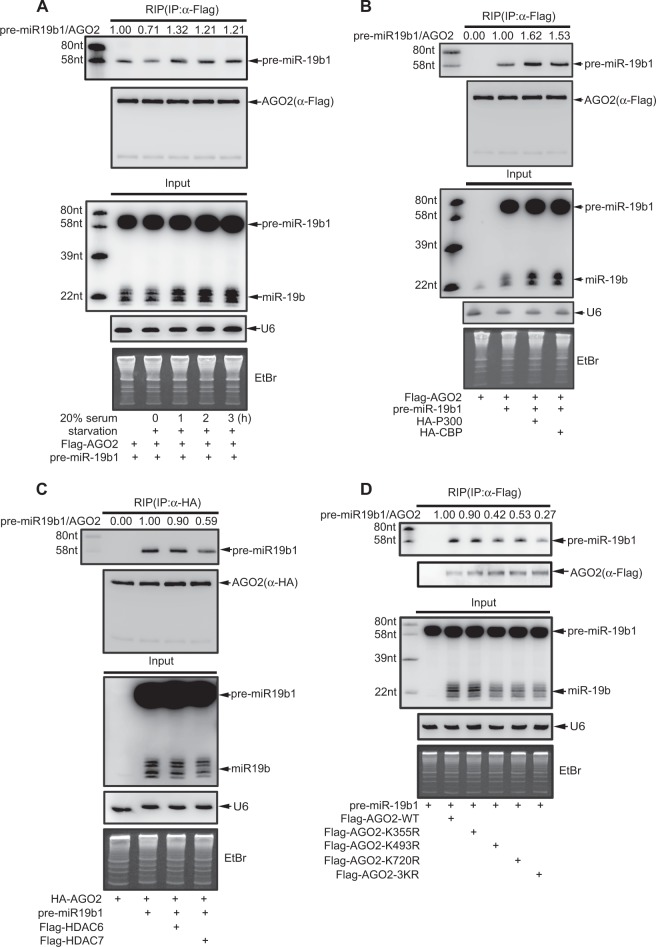

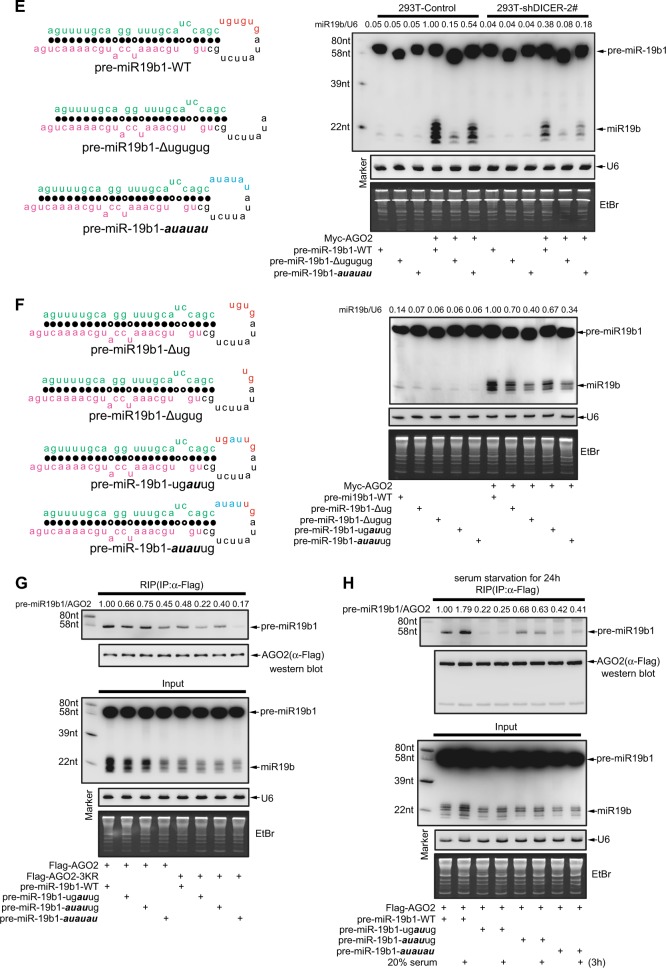
**a**–**d** AGO2 acetylation enhances its binding with pre-miR-19b1. **a** Serum stimulates pre-miR-19b1 binding to AGO2. 293T cells were co-transfected pre-miR-19b1 with Flag-AGO2 for 20 h, then were serum-starved for 24 h, and followed by stimulation with 20% serum for indicated times. Then cells were lysed by RIP lysis buffer for the RIP assay with anti-Flag antibody, followed by northern blotting analysis. **b** P300/CBP enhances the interaction between pre-miR-19b1 and AGO2. Pre-miR-19b1 and Flag-tagged AGO2 were co-transfected with HA-P300 or HA-CBP into 293T cells, respectively. The RIP/northern blotting analysis were performed as before. **c** HDAC7 but not HDAC6 reduces the interaction between pre-miR-19b1 and AGO2. HA-tagged AGO2 and pre-miR-19b1 were co-transfected with Flag-HDAC6 or Flag-HDAC7 into 293T cells. The RIP/northern blotting analysis were performed as before. **d** Acetyl-mutants K493R or K720R, but not K355R of AGO2 decreases its interaction with pre-miR-19b1. AGO2-WT and mutants AGO2-K355R, -K493R, -K720R and -3KR were individually co-transfected with pre-miR-19b1 into 293T cells. 48 h later, The RIP/northern blotting analysis was performed as before. The efficiencies of RNA immunoprecipitation and mature miR-19b in Input were determined by WB Northern blotting, respectively, in all above experiments. **e**–**h** The UGUGUG Motif in the Terminal Loop of pre-miR-19b1 is a specific processing feature for the interaction with acetylated AGO2. **e**, **f** Deletion or mutation of the UGUGUG motif in the terminal loop of pre-miR-19b1 impairs pre-miR-19b1 processing mediated by AGO2 in a DICER dependent manner. **e** pre-miR-19b1-WT, pre-miR-19b1-∆UGUGUG or pre-miR-19b1-*AUAUAU* was co-transfected with Myc-AGO2 into 293T-shControl or 293T-shDICER cells; (**f**) pre-miR-19b1-WT, pre-miR-19b1-∆UG, pre-miR-19b1-∆UGUG, pre-miR-19b1-UG*AU*UG or pre-miR-19b1-*AUAU*UG were co-transfected with Myc-AGO2 into 293T cells, 48 h later, the total RNAs were extracted and followed by northern blotting analysis. **g** The UGUGUG motif of pre-miR-19b1 is necessary for its interaction with acetylated AGO2. Pre-miR-19b1-WT, pre-miR-19b1-UG*AU*UG, pre-miR-19b1-*AUAU*UG and pre-miR-19b1-*AUAUAU* were co-transfected with Flag-AGO2-WT or -3KR into 293T cells. 48 h later, cells were lysed by for the RIP assay with anti-Flag antibody, the interaction of pre-miR-19b1 with Flag-AGO2-WT or Flag-AGO2-3KR was detected by northern blotting analysis. **h** Serum stimulation enhances the association of AGO2 with pre-miR-19b1-WT, but not the UGUGUG-motif mutated pre-miR-19b1. Pre-miR-19b1-WT, pre-miR-19b1-UG*AU*UG, pre-miR-19b1-*AUAU*UG or pre-miR-19b1-*AUAUAU* were co-transfected with Flag-AGO2 into 293T cells for 20 h, then were serum-starved for 24 h, and followed by stimulation with 20% serum for for 3 h. The RIP/northern blotting analysis was performed as before. The efficiencies of RNA immunoprecipitation and mature miR-19b in input were determined by WB and Northern blotting, respectively

Next, we performed an RNA immmunoprecipitation (RIP) assay with the same experiment conditions as Fig. 4d–g to validate the hypothesis that acetylation of AGO2 is necessary for its interaction with pre-miR-19b1 and follow-up miR-19b maturation processing. Firstly, AGO2 and pre-miR-19b1 were co-transfected into 293 T cells, and then followed by serum starvation for 24 h and re-treatment with 20% serum for indicated times. As expectedly, the RIP result showed that AGO2 recruiting pre-miR-19b1 was increased by serum stimulation (Fig. 5a). Further, we confirmed that the capability of AGO2 recruiting pre-miR-19b1 was enhanced by the acetyltransferases P300 and CBP (Fig. 5b), on the contrary, decreased by the deacetylase HDAC7 but not HDAC6 (Fig. 5c). Lastly, we found that the binding of acetyl-mutants AGO2-K493R, -K720R or -3KR with pre-miR-19b1 was attenuated, but not AGO2-K355R, compared to that of AGO2-WT (Fig. 5d). Meanwhile, the mature miR-19b productions regulated by AGO2 were experimentally repeated, as shown in Input panels (Fig. 5a–d). Collectively, these results suggest that AGO2 acetylation regulates the capacity of AGO2-recruiting pre-miR-19b1, thus to control the production of mature miR-19b.

### The UGUGUG motif in the terminal loop of pre-miR-19b1 is a specific processing feature for the interaction with acetylated AGO2

The motifs on the stem-loop-structured miRNA-precursors are critical for its maturation processing, which is regulated by many RNA binding proteins (RBPs) [31]. Some specific motifs can be recognized certain RBPs, for an example, the GU/UG motif in the pre-miR-9 is specifically bound by LIN28A and thus to suppress its processing [32]. We noticed a three tandem UG repeats, UGUGUG, in the terminal loop region of pre-miR-19b1, and wondered whether it is a specific processing feature recognized and bound by acetylated AGO2. To this end, we deleted the motif UGUGUG or mutated it to ***AUAUAU*** in pre-miR-19b1 (Fig. 5e, left panels) and transfected them with or without AGO2 into 293T-shControl or 293T-shDICER stable cell lines, respectively, then followed by Northern blotting analysis. Without co-transfection of AGO2, the biogenesis of mature miR-19b from three pre-miR-19b1 forms including the WT, the (UG)_3_-deleted (-∆UGUGUG) and the (UG)_3_-mutated (-***AUAUAU***) was all the same low level. However with co-transfection of AGO2, the biogenesis of mature miR-19b produced from the (UG)_3_-deleted or (UG)_3_-mutated forms of pre-miR-19b1 was almost abolished or significantly decreased, respectively, when compared to that of in the WT form in both cell lines, and the whole biogenesis of mature miR-19b in 293T-shDICER cells was much lesser than that of in 293T-shControl cells (Fig. 5e, right panels). These results suggested that the motif UGUGUG of pre-miR-19b1 plays a great role in its processing in a DICER dependent manner. To further investigate whether each UG of this three-tandem repeat in the terminal loop of pre-miR-19b1 is required for its binding to AGO2, we also generated single/double UG-deleted or UG-mutated forms including pre-miR-19b1-∆UG, -∆UGUG, -UG***AU***UG and -***AUAU***UG (as indicated in Fig. 5f, left panels), and co-transfected them with or without AGO2 into 293 T cells. Compared with pre-miR-19b1-WT, the levels of mature miR-19b from pre-miR-19b1-∆UG, -∆UGUG, -UG***AU***UG and -***AUAU***UG mediated by AGO2 were greatly decreased; meanwhile, mature miR-19b from (UG)_2_-deleted/mutated (-∆UGUG and -***AUAU***UG) were much lesser than that from (UG)_1_-deleted/mutated (-∆UG and -UG***AU***UG) (Fig. 5f, right panels). Collectively, these results demonstrate that the three-tandem UG repeat is essential for AGO2-mediated processing of mature miR-19b from pre-miR-19b1.

To validate the hypothesis that the UGUGUG of pre-miR-19b1 is a key motif for the interaction with acetylated AGO2 to regulate miR-19b biogenesis, we co-transfected pre-miR-19b1-WT or three mutants pre-miR-19b1-UG***AU***UG, -***AUAU***UG and -***AUAUAU*** with AGO2-WT or AGO2-3KR into 293 T cells, and performed the RIP assays. Indeed, the result showed that the binding of three mutants pre-miR-19b1-UG***AU***UG, -***AUAU***UG and -***AUAUAU*** to AGO2-WT were all less than that of pre-miR-19b1-WT (Fig. 5g, lanes 1-4). As a meanwhile, the patterns of four pre-miR-19b1 forms recruited by AGO2-3KR were similar to those by AGO2-WT, however the levels were much lower (Fig. 5g, lanes 5–8). Moreover, these four pre-miR-19b1 forms were co-transfected with AGO2 into 293 T cells, and then followed by serum starvation for 24 h and serum stimulation as indicated time, which has been proved to enhance AGO2 acetylation (Fig. 2a). The RIP assay result showed that serum stimulation greatly increased the association between pre-miR-19b1-WT with AGO2, but not the interactions between three mutants pre-miR-19b1-UG***AU***UG, -***AUAU***UG and -***AUAUAU*** with AGO2 (Fig. 5h). This result suggested that the UGUGUG motif of pre-miR-19b1 was essential for its binding to acetylated AGO2. Collectively, above results reveal that the motif UGUGUG in the terminal loop of pre-miR-19b1 is a specific processing feature recognized and bound by acetylated AGO2 and is essential for miR-19b biogenesis.

### Acetylation of AGO2 promotes cancer progression via the miR-19b pathway

**Fig. 6:**
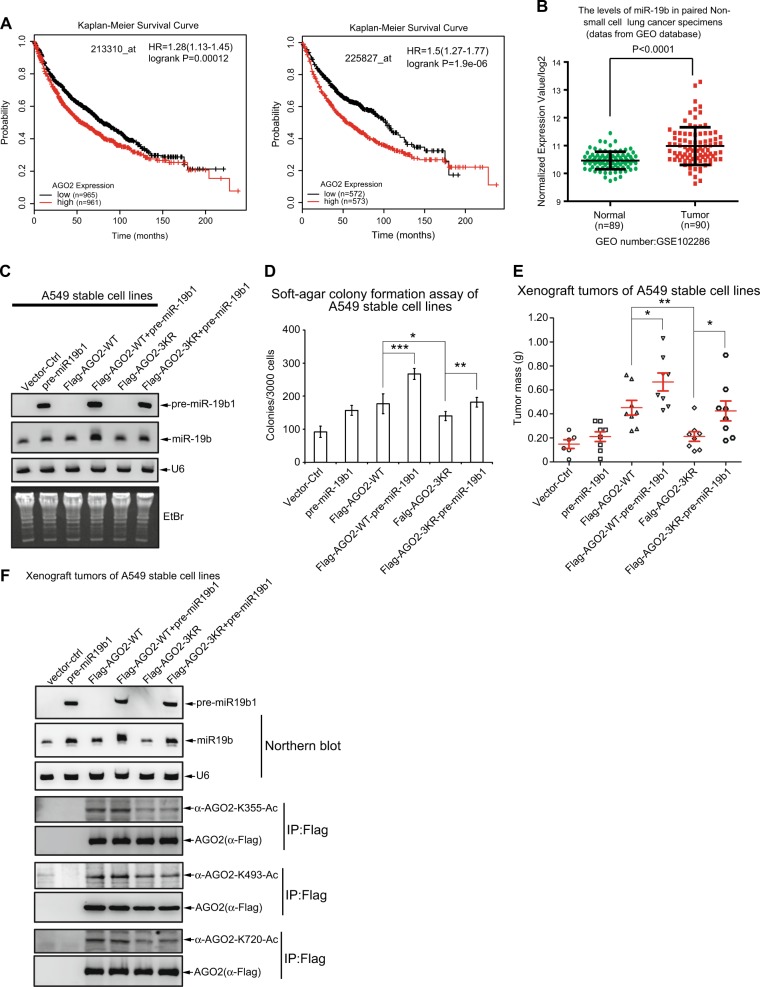
AGO2 acetylation promotes cancer progression *via* the miR-19b pathway. **a** The correlation between the AGO2 expression level and the survival of lung cancer patients from GEO database was analyzed by the Kaplan–Meier method. **b** The miR19b expression levels in normal lung tissues (*n* = 89) and paired clinical lung specimens tumor (*n* = 90) from GEO database (GSE102286) were analyzed. **c** The expression levels of pre-miR-19b1 and mature miR-19b1 in A549 stable cell lines were determined by northern blotting. See Figure S7A for the expression levels of AGO2-WT, AGO2-3KR in A549 stable cell lines were comparable. **d** Acetyl-mutations of AGO2 inhibit the soft agar colony formation. 3 × 10^3^ of above A549 stable cell lines were seeded in 2 ml of medium containing 5% FBS with 0.35% agar. Colonies were stained with 0.005% crystal violet three weeks later, photos were taken and the number of colonies was scored. *P* values of < 0.05 (*), < 0.01 (**), < 0.001 (***). **e** AGO2 acetylation increases xenografted tumor growth. 2.5 × 10^6^ of above A549 stable cell lines were injected subcutaneously into the back of nude mice. At 35 days tumors were dissected and assessed by weight. See Figure S7B for the sizes of xenografted tumors. **f** Verification for the expression levels of pre-miR-19b1, mature miR-19b1 and AGO2 acetylaion levels in xenograft tumors with A549 stable cell lines. Total RNAs were extracted from xenograft tumors and followed by northern blotting analysis for pre-miR-19b1 and mature miR-19b1. Lysates from xenograft tumors were used for IP with anti-Flag antibody, and then were detected by AGO2 specific acetyl-antibodies. See Figure S7C for the expression levels of AGO2-WT, AGO2-3KR in xenograft tumors were comparable

By employing public clinical data, the Kaplan–Meier survival analysis revealed that lung cancer patients with the high levels of AGO2 had a lower survival rate than those with the low levels of AGO2 (Fig. 6a), suggesting that the high expression levels of AGO2 may be a risk factor of lung cancer. MiR-19b, as the core component of miR-17-92 cluster, is related with and highly expressed in many diseases and cancers [6, 7]. As expectedly, we found that the expression levels of miR-19b in clinical lung cancer specimens were much higher than those in paired normal tissues (GEO database GSE10228) (Fig. 6b). These results implied that both AGO2 and miR-19b may correlate with lung cancer progression.

Next, to test our hypothesis that acetylation of AGO2 promotes tumorigenesis by specifically increasing mature miR-19b, we used a lung carcinoma cell line A549 to generate stable cell lines expressing the control vector, pre-miR-19b1, AGO2-WT, AGO2-WT-pre-miR-19b1, AGO2-3KR and AGO2-3KR-pre-miR-19b1. The expression levels of AGO2 were detected by immunoblotting with anti-AGO2 or anti-Flag antibody, confirming AGO2-WT, -3KR comparable in corresponding cells (Figure S7A). In the meanwhile, the expression levels of mature miR-19b and pre-miR-19b1 were analyzed by northern bloting, validating that AGO2-WT other than AGO2-3KR significantly increased mature miR-19b in those cells (Fig. 6c). To explore whether AGO2 acetylation affects the transforming potential, we performed a soft agar colony-forming assay of each stable A549 cell lines. Cells transfected with either pre-miR-19b or AGO2-WT showed promotion of colony growth when compared with the control vector transfected cells. AGO2-WT together with pre-miR-19b1 (AGO2-WT-pre-miR-19b1) greatly increased anchorage-independent colony formation and growth compared to those of cells transfected with only AGO2-WT or pre-miR-19b1, whereas the acetylation mutant AGO2-3KR appeared to lose this capacity (Fig. 6d). Furthermore, to investigate whether the increased miR-19b by AGO2 acetylation also influences tumor growth in vivo, each of stable A549 cell lines was subcutaneously inoculated into the backs of nude mice. The xenograft tumor growth (Fig. 6e, S7B) with these stable cell lines (Figure S7C) showed the very similar pattern of results as those in the soft agar colony-forming assays. Especially, tumors in the AGO2-WT-pre-miR-19b1 group exhibited distinct promotion of growth compared to those in the control vector, AGO2-WT, or pre-miR-19b1 group, indicating that the increased miR-19b mediated by AGO2 acetylation leads to the tumor growth. However, tumors in the AGO2-3KR or AGO2-3KR-pre-miR-19b1 group grew much more slowly than those in the AGO2-WT or AGO2-WT-pre-miR-19b1 group (Fig. 6e), respectively, suggesting the acetyl-mutant AGO2-3KR lost its function in promoting tumor growth. The growth patterns of xenograft tumors were highly related with the expression levels of miR-19b, which were controlled by the status of AGO2 acetylation (Fig. 6f). Taken together, these results demonstrate that acetylation of Ago2 is required for its roles in promoting tumorigenesis by increasing miR-19b biogenesis.

### AGO2 acetylation is up-regulated and positively correlated with miR-19b expression in human lung cancers

To determine the pathological relevance of miR-19b expression regulated by AGO2 acetylation at K493 and K720 in human cancers, we performed ISH staining to determine the expression levels of miR-19b and IHC staining for the acetylation levels of AGO2-K493-Ac and AGO2-K720-Ac in the same lung cancer tissue arrays. The clinical pathological characteristics of these samples in this study were summarized (Figure S8A). Significantly, we observed that the expression levels of miR-19b were obviously increased in lung cancers compared with normal tissues (*P* < 0.0001, unpaired *t*-test), and the acetylation levels of AGO2-K493-Ac (*P* = 0.0142, unpaired *t*-test) and AGO2-K720-Ac (*P* = 0.001, unpaired *t*-test) were also significantly higher in tumors than those in normal tissues (Figure S8B-D). These findings suggest that miR-19b may be upregulated and correlated with high acetylation levels of AGO2-K493-Ac and AGO2-K720-Ac in human lung cancers.

**Fig. 7:**
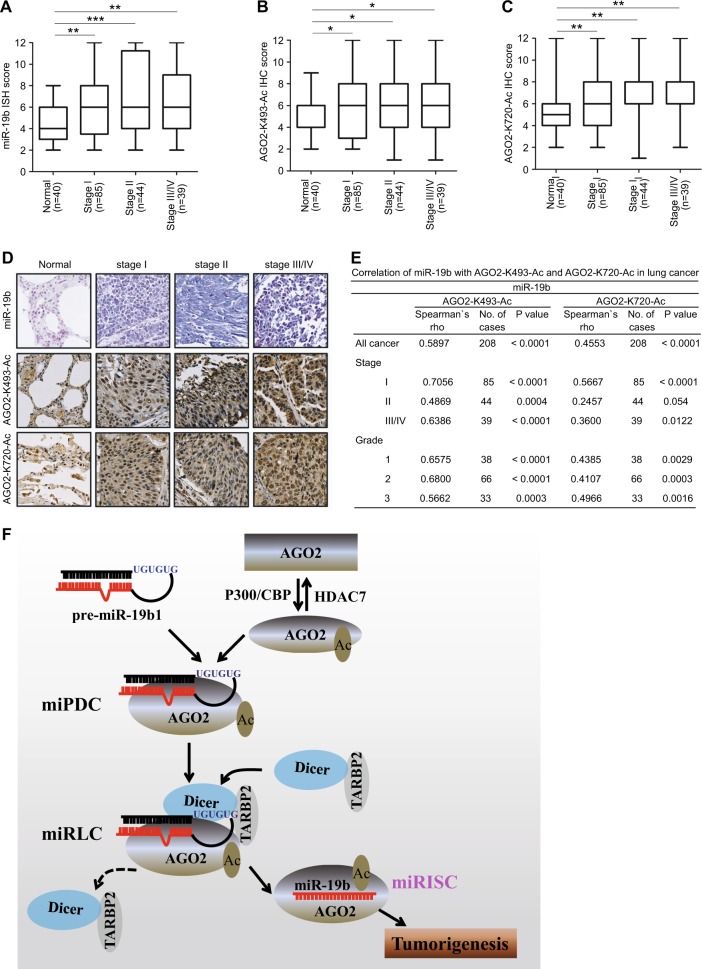
AGO2 acetylation is upregulated and positively correlated with miR-19b expression in human lung cancers. **a**–**c** The levels of miR-19b1, AGO2-K493-Ac and AGO2-K720-Ac in pathological sub-stage lung cancers were much higher than those in normal tissues. ISH staining scores for miR-19b (**a**) and IHC staining scores for AGO2-K493-Ac (**b**) and AGO2-K720-Ac (**c**) in normal tissues and pathological sub-stage lung cancers were shown. ISH and IHC scores were compared by one-way ANOVA and an independent *t*-test. The error bars represent mean ± s.d., *P* values of < 0.05 (*), < 0.01 (**), < 0.001 (***). **d** Representative images of ISH staining for miR-19b and IHC staining for AGO2-K493-Ac and AGO2-K720-Ac in normal tissues and pathological sub-stages lung cancer tissues. **e** The expression levels of miR-19b are positively correlated with the acetylation levels of AGO2-K493-Ac and AGO2-K720-Ac in lung cancers. Data were analyzed using a one-way ANOVA and independent *t*-test. **f** A novel mechanism that AGO2 acetylation promotes tumorigenesis by enhancing miR-19b maturation

With one-way ANOVA analysis and independent *t*-test, we showed that the expression levels of miR-19b had statistically significant differences between normal tissues with pathological sub-stage-groups (*P* = 0.0011). We also observed very similar results for the acetylation levels of AGO2-K493-Ac (*P* = 0.1082) and AGO2-K720-Ac (*P* = 0.0097) (Fig. 7a–c, Figure S8A). Representative images of ISH staining for miR-19b and IHC staining for AGO2-K493-Ac and AGO2-K720-Ac in normal tissues and pathological sub-stage lung cancer tissues were shown (Fig. 7d). Most importantly, in comparison of all normal tissues and lung cancers, the expression of miR-19b was positively correlated with the expression of AGO2-K493-Ac (spearman’s rho = 0.5897, *P* < 0.0001) and AGO2-K720-Ac (spearman’s rho = 0.4553, *P* < 0.0001), respectively (Fig. 7e). Moreover, the expression levels of miR-19b were positively correlated with the acetylation levels of AGO2-K493-Ac in pathological stages (Stage I, spearman’s rho = 0.7056, *P* < 0.0001; Stage II, spearman’s rho = 0.4869, *P* < 0.0004; Stage III/IV, spearman’s rho = 0.6386, *P* < 0.0001) and tumor grades (Grade 1, spearman’s rho = 0.6575, *P* < 0.0001; Grade 2, spearman’s rho = 0.68, *P* < 0.0001; Grade 3, spearman’s rho = 0.5662, P < 0.0003) (Fig. 7e). Simultaneously, the expression levels of miR-19b were also positively correlated with the acetylation levels of AGO2-K720-Ac in pathological stages (Stage I, spearman’s rho = 0.5667, *P* < 0.0001; Stage II, spearman’s rho = 0.2457, *P* = 0.054; Stage III/IV, spearman’s rho = 0.36, *P* = 0.0122) and tumor grades (Grade 1, spearman’s rho = 0.4385, *P* = 0.0029; Grade 2, spearman’s rho = 0.4107, *P* = 0.0003; Grade 3, spearman’s rho = 0.4966, *P* = 0.0016) (Fig. 7e). These results suggest that the high levels of miR-19b, AGO2-K493-Ac and AGO2-K720-Ac are significantly associated with lung cancer progression. Taken together, above findings demonstrate that the high acetylation levels of AGO2 at K493 and K720 are positively correlated with miR-19b expression in lung cancers, revealing a novel mechanism that AGO2 acetylation promotes tumorigenesis by enhancing miR-19b maturation.

## Discussion

In this work, as summarized in Fig. 7f, we identify a new PTM of AGO2, acetylation, by mass spectormetry analysis, point-mutation biochemical approaches and home-made specific acetyl-antibodies. We provide both direct evidence for AGO2 acetylation by the acetyltransferases P300/CBP and its deacetylation by the deacetylase HDAC7. We demonstrate that acetylation of AGO2 increases its recruiting pre-miR-19b1 to form the miPDC (AGO2-pre-miR-19b1), thereby to enhance miR-19b maturation. Most importantly, the motif UGUGUG in the terminal loop of pre-miR-19b1, as a specific processing feature that is recognized and bound by acetylated AGO2, is essential for the miRLC assembly. By analyses on public clinical data, xenograft mouse models, and IHC and ISH staining of lung cancer tissue arrays, we confirm that AGO2 acetylation promotes cancer progression by increasing miR-19b maturation. Thus, our results provide crucial insight into how miR-19b maturation is regulated by AGO2 acetylation and why miR-19b is highly expressed in lung cancer.

AGO2 acts as an efficient modulator in regulating miRNA processing, miRNA-guided gene silencing and other functions through various PTMs, including prolyl-4-hydroxylation [20, 21], phosphorylation [22–26], ubiquitination [27, 33], polyADP-ribosylation [28] and SUMOylation [34]. Our study for the first time identified that AGO2 was acetylated. Diverse external stimuli and specific environmental conditions can dynamically regulate PTMs of AGO2 [21, 23, 33, 35]. We found that the acetylation of AGO2 induced by P300 under serum stimulation. Thus, in responses to various intercellular and extracellular environment stimuli, AGO2 seems to be accurately modified with different PTMs and as an intercellular mediator elaborately modulates the miRNA pathways in various kinds of biological processes.

Prior to the formation of miRLC, some pre-miRNAs can be directly recruited by AGO2 to form a specific complex miPDC [18, 19, 36]. However, the detailed mechanism for miPDC assembly remains unclear. Our mechanistic studies with the RIP assays showed that enhanced acetylation of AGO2 by both serum stimulation and P300/CBP coexpression increased its interaction with pre-miR-19b1. Our results revealed that AGO2 acetylation at K493 and K720 potentially modulated the formation of miPDC comprised of AGO2 and pre-miR-19b1. The acetylation sites K493 and K720 of AGO2 were located at its MID and PIWI domains, respectively, thus we speculated that AGO2 acetylation at K493 and K720 perhaps may alter its structure, specifically influencing its interaction with pre-miR-19b1. Although it has been reported the structure of human AGO2 in complex with mature miR-20a [37], the crystal structure of miPDC complex of AGO2 with certain pre-miRNA has not been resolved until now. Further work will be required to unveil the mechanism underlying how pre-miRNA interacts with acetylated AGO2 on crystal structure level.

The Mourelatos lab found that a miPDC not only plays a critical role in the maturation of the DICER-independent miR-451, but also enhances the expression of certain DICER-dependent miRNAs by bringing them to the miRLC for DICER-directed processing. Remarkably, preferable features of certain pre-miRNAs assembled into miPDC bear a similarity to the the characteristics: 5’-uridine, and 3’-mid base pairing and 5’-mid mismatches in the stem-region [19]. However, whether a specific feature in the terminal loop of pre-miRNAs promotes miPDC assembly is not explored. More recently the Meister lab has identified large number of RNA binding proteins (RBPs) specifically interacting with distinct pre-miRNAs by using a proteomics-based pull-down approach, and found that these RBPs of pre-miRNAs modulate levels of mature miRNAs [31]. Notably, both loop and stem regions of pre-miRNAs serve as determinants of miRNA hairpin recognition by RBPs. Moreover, the Bartel lab found that in addition to secondary structure of pri-miRNA hairpins, primary-sequence motifs including the basal UG and CNNC, and the GUG in the loop and apical stem (for example, a loop-binding motif UGUG in pre-miR-30a) also contribute to efficient processing in human cells [38]. Moreover, the GU-rich region (UGGUGUGG) in the terminal loop/apical stem of pre-miR-9 bound to LIN28A was identified by footprint analysis [32]. These suggest that the GU/UG-rich motif in the conserved terminal loop of pri/pre-miRNAs possesses the capacity for determination of its processing. In this study we have identified that the UGUGUG motif in the terminal loop (apical stem) of pre-miR-19b1 plays an essential role for its association with ac-AGO2 and subsequently promoting miR-19b1 maturation.

MiR-17-92 cluster, as a firstly identified ‘*oncomiR*’, is positively correlated with B-cell lymphoma tumor developing [3]. Among the six miRNAs of miR-17-92, miR-19a and miR-19b are key oncogenic components, which sufficiently recapitulate the oncogenic properties of the entire cluster [6, 7]. With biochemical, cellular and clinical approaches, we have systemically demonstrated that acetylation of AGO2 was essential for pre-miR-19b1 processing into mature miR-19b, which are proven to promote cancer progression. Our data discovered that AGO2 acetylation have a critical role in accelerating tumorigenesis, and provided a potential therapeutic strategy for patients with the high acetylation level of AGO2.

Based on our new finding, here we reviewed and proposed an integrated model for pre-miRNA programmed silencing complex assembly pathways in mammals: (a) In the canonical pathway, a pre-miRNA exported from the nucleus is directly recruited to a performed complex of DICER/TARBP2/AGO2 to constitute miRLC [19, 39–42] for excision by DICER into an asymmetrical miRNA/miRNA* duplex intermediate [43–45], which is sub-sequentially loaded onto AGO2 for the single-stranded miRNA maturation [46, 47]. The canonical pathway is previously believed to be universal to most miRNAs, however certain pre-miRNAs are firstly recruited by AGO2 to form AGO2:pre-miRNA [18] or miPDC [19]. There are four pathways of miRNA maturation via miPDC as followed. (b) As a prominent example, pre-miR451, which is bound to AGO2 to form a miPDC, is sliced by AGO2 with its endonuclease activity into an approximately 30-nt cleaved intermediate in an alternative DICER-independent way [14–16, 19]. Subsequently, the 3’ end of the AGO2-cleaved pre-miR-451 intermediate is trimmed to the mature length by poly(A)-specific ribonuclease (PARN) [48]. (c) A subset of pre-miRNAs, such as pre-let-7a, pre-miR-16 and pre-miR-21, are firstly cleaved by AGO2 itself at the pre-miRNA hairpin 12 nucleotides from its 3’-end into an additional processing intermediates, called as AGO2-cleaved precursor miRNAs (ac-pre-miRNAs), and then they were eventually cleaved by DICER for maturation [17]. (d) Another subset of pre-miRNAs are also directly recruited by AGO2 to form miPDC, in which pre-miRNAs are not cleaved by AGO2. These miPDCs assemble with DICER/TARBP2 complex into miRLC, and followed by Dicer-dependent pre-miRNA processing into mature miRNAs [19, 36]. (e) In this study, we found that a novel pathway of miR-19b1 maturation is similar to (d) in DICER-dependent manner, however the miPDC assembly of pre-mi19b1 to AGO2 is dependent on the UGUGUG motif in the terminal loop of pre-miR-19b1 and specifically enhanced by AGO2 acetylation (Fig. 7f).

## Methods

### Cell cultures and transfection

Human embryonic kidney 293 T, 293FT, HeLa and A549 cells were cultured in Dulbecco’s modified Eagle’s medium (DMEM, Hyclone) containing 10% fetal calf serum (FBS, Biowest), 1% penicillin-streptomycin (Invitrogen) at 37 °C with 5% CO_2_. All transfections were performed by using lipofectamine 2000 (Invitrogen).

### Antibodies and reagents

Antibodies against AGO2 (#2897; working dilution with 1:1000), acetylated lysine (#9441 s; working dilution with 1:1000), DICER (#3363; working dilution with 1:1000), Myc (#2276; working dilution with 1:1000) and GFP (#2956; working dilution with 1:2000) were from Cell Signaling Technology. Monoclonal anti-Flag M2 (#F1804; working dilution with 1:1000) and anti-HDAC7 (#KG-17; working dilution with 1:1000) were from Sigma. Monoclonal anti-AGO2 (ab57113; working dilution with 1:1000) was from abcam. Monoclonal anti-HA (#A448-101L; working dilution with 1:1000) was from Covance. Antibody against P300 (C-20; working dilution with 1:200), normal mouse IgG sc-2025 (#J1810; working dilution with 1:250) and normal rabbit IgG sc-2027 (#I2310; working dilution with 1:250) were from Santa Cruz Biotechnology. Anti-GAPDH (#60004-1-Ig; working dilution with 1:2000) and anti-Tubulin (#66031-1-Ig; working dilution with 1:2000) were from Protein Tech Group. Antibody against GST (#CW0084; working dilution with 1:2000) was from CWbioTech (Shanghai, China). Protein G Plus/Protein A agarose suspension (#IP05) was purchased from Calbiochem. Glutathione Sepharose 4B (#17-0756-01) was from GE Healthcare Life Sciences (USA). Deacetylase inhibitors TSA and NAM, polybrene (hexadimethrine bromide; #H9268) and puromycin (#P8833) were from Sigma.

Antibodies against AGO2 specific acetyl-K355, -K493 and -K720 were generated by immunizing rabbits with antigen peptides at Shanghai HuiOu biotechnology Co. Ltd. Briefly, chemical synthesized acetyl-peptides and non-acetyl-peptides as below: K355-Ac peptide (C-AGQRCIKK(Ac)LTD), K355 peptide (C-AGQRCIKKLTD), K493-Ac peptide (QGQPCFCK(Ac)YAQ), K493 peptide (QGQPCFCKYAQ), K720-Ac peptide (C-TRLFCTDK(Ac)NER) and K720 peptide (C-TRLFCTDKNER). An additional cysteine at the N-terminal of peptides was added for following KLH (Keyhole limpet hemocyanin) conjugation. These AGO2 specific acetyl-peptides and non-acetyl-peptides were conjugated with KLH as antigens. The new zealand white rabbit were subcutaneously injected with the antigens in different places at 0 day, 30 days, 60 days and 90 days, respectively. Furthermore, 15 days later, blood was collected from rabbit carotid for antibodies potency detection by ELISA assay. Finally, the AGO2 specific acetyl-antibodies were firstly filtered by non-acetyl-peptides antigen column, and secondly purified with acetyl-peptides antigen column.

### Plasmids

The plasmid pCS2-MYC_6_-AGO2 [49] was gifted from Dr Gunter Meister, Universität Regensburg. Point mutations of AGO2 were generated by using the KOD-plus-mutagenesis Kit (TOYOBO). The full-length and acetylation-site mutated AGO2 were cloned into the lentiviral expression vector CD513B for establishing stable cell lines with the packaging plasmids pMD2G and pCMV-dR8. The AGO2 cDNA was cloned from pCS2-Myc_6_-AGO2 into the vector pGEX-4T-1 (GE healthcare) for the expression of GST-AGO2 fusion protein. The expressing constructs HA-P300 and HA-CBP were previously described [30]. Flag-Sirt1、Flag-Sirt5 and Flag-HDAC7 were kindly provided by Dr JK Cheng at Shanghai Jiao Tong University School of Medicine. The AGO2 shRNA oligo sequences were gained from Sigma and sub-cloned into the lentiviral vector pLKO.1. The precursor miRNAs pre-miR-19b1 and pre-miR-19b2 were cloned into the pGREENpuro vector. Mutations of pre-miR-19b1 were sub-cloned by using the KOD-plus-mutagenesis Kit (TOYOBO). Primer sequences for construction plasmid and shRNA sequences were listed in Supplementary Table S2.

### In vitro GST-AGO2 acetylation or deacetylation assays

The prokaryotic expression vector pGEX-4T-1-AGO2 was transformed into BL21 competent cells with 0.5 mM IPTG inducing for 16 h at 16 °C. Bacterial cells were lysed in B-PER Protein Extraction Reagent (#78248, Thermo Fisher, USA) with 100 μg/ml lysozyme (Sigma), 12.5 μl/ml protease inhibitor cocktails (Sangon) and 2 U/ml DNaseI (Thermo Fisher) for 1 h at room temperature. The fusion protein GST-AGO2 was purified with Glutathione sepharose 4B beads (GE healthcare) and gradually eluted with 20 mM GSH (reduced glutathione; 50 mM Tris pH8.0) and 10 mM GSH for 15 minutes. For in vitro GST-AGO2 acetylation or deacetylation assays, HEK293T cells were transfected with indicated plasmids or treatment as indicated conditions, then cells were lysed with RIPA buffer (50 mM Tris-HCl pH7.4, 150 mM NaCl, 1% NP-40, protease inhibitor cocktail (Roche)) on ice for 1 h. And then lysates were incubated with 10 μg of GST-AGO2 and 25 μl of Glutathione sepharose 4B beads at 4 °C overnight and then incubated at 30 °C for 0.5 h. GST-AGO2 bound to beads were washed for five times by using the same RIPA buffer, and followed by Western blotting analysis through the indicated antibodies.

### Co-Immunoprecipitation (Co-IP)

HEK293T or HeLa cells transfected with indicated plasmids were lysed in RIPA buffer (50 mM Tris-HCl pH7.4, 150 mM NaCl, 1% NP-40, protease inhibitor cocktail (Roche)) on ice for 1 h. For Co-immunoprecipitation (Co-IP), 500~1000 μg of total lysed proteins and 25 μl protein A/G-agarose beads (Calbiochem) were incubated with indicated antibodies at 4 °C overnight, and then washed for five times with RIPA buffer without protease inhibitor cocktail and followed by Western blotting analysis.

### Acetylation analysis by mass spectrometry

HEK293T cells were transfected with Flag-AGO2 for 48 h, and then were treated with 2 μM TSA for 6 h and 10 mM NAM for 18 h, respectively, before harvested. Cells were lysed with the RIPA buffer (50 mM Tris-HCl pH7.4, 150 mM NaCl, 10 mM EDTA, 0.1% SDS, 1% NP-40, protease inhibitor cocktail tablet), and lysates were incubated with monoclonal Flag antibody and protein A/G-agarose beads at 4 °C overnight. Beads were washed for five times with the same RIPA buffer, and 5% of beads were subjected to SDS-Polyacrylamide gels (8%) for Western blotting analysis. 95% of beads were identified with Coomassie blue staining, Flag-AGO2 bands were cut, and digested with trypsin for acetylation analysis of Q Exactive Mass Spectrometer.

### Small RNA high-throughput sequencing and analysis

The method for small RNA high-throughput sequencing was previously described [50]. Briefly, small RNA (sRNA) population was isolated by separating total RNAs extracted from A549 stable cell lines on denaturing polyacrylamide gel electrophoresis (PAGE), and cut a portion of the gel corresponding to the size 18–30 nucleotides based standard oligonucleotide markers. 3’-adapter was ligated to sRNA population and ligated RNAs (36–50 nt) were purified by running on urea PAGE. This was followed by 5’-adapter ligation and purification of adapter ligated RNAs (62–75 nt) in a similar manner. Modified sRNAs were reversely transcribed and then PCR amplified with adapter specific primers and the amplified cDNAs were finally purified on Urea PAGE to generate cDNA tag libraries for sequencing by Illumina Hiseq 2000.

The sequence tags from Hiseq sequencing went through the data cleaning analysis to get credible clean tags. Then the standard bioinformatics analysis was performed to annotate the clean tags. Tags alignment to miRBase database by using blast or bowtie to identify known miRNA; sRNA mapping to repeat with processes to screen and remove repeat associated reads; Tags alignment to Genbank and Rfam database with blast or bowtie to screen and remove rRNA, scRNA, snoRNA, snRNA, tRNA associated reads. Comparing the known or novel miRNA expression between two samples to find out the differentially expressed miRNA as following procedure: (a) normalize the expression of miRNAs in two samples (control and treatment) to get the expression of transcript per million (TPM). The normalization formula is that normalized expression = actual miRNA count/total count of clean reads × 1,000,000. (b) Calculate fold-change from the normalized expression then generate the log2 ratio plot and scatter plot. The fold-change formula is that Fold_change = log2 (treatment/control).

### Northern blotting analysis

Total RNAs extracted by TRIZOL reagent (Invitrogen) from cells or immunoprecipitated beads were denatured and fractionated by electrophoresis on a 20% polyacrylamide-8M urea gel, then transferred the RNAs to a nylon membrane (Roche) and cross-linked, then dealing with following two methods: (1), Pre-hybridized the membrane with DIG Easy Hyb:Formamide Deionized (1:1) at 42 °C for 30 min, and then hybridized with DIG-labeled DNA probe within DIG Easy Hyb:Formamide Deionized (1:1) at 42 °C overnight. The membrane was washed with 2 × SSC and 0.1 × SSC-0.1% SDS buffer at 37 °C, each buffer for twice times. The membrane was incubated with an anti-digoxigenin antibody (Roche) and then incubated with CDP-Star (ABI), the RNA was detected on X-ray film. (2), The membrane was pre-hybridized by North2South® Hybridization Buffer at 55 °C for 30 min, and then was hybridized with biotinylated probe within North2South® Hybridization Buffer at 55 °C overnight; and following washed with North2South® Hybridization Stringency Wash Buffer (1×) 15 min at 55 °C for twice times, then incubated within streptavidin-HRP soluted blocking Buffer for 1 h at room temperature, and washed the membrane by wash buffer 5 min for four times and by substrate equilibration buffer 5 min for once, respectively. Finally, the signaling on membrane was detected by using Amersham Imager 600 (GE) instrument.

### qRT-PCR analysis

The method for miRNA qRT-PCR was previously described with modifications [50, 51]. Briefly, extracted RNAs by 1 ml of TRIZOL reagent (Invitrogen) were instantaneously treated with DNaseI (Thermo) to degrade genomic DNA. Reverse transcription was performed by using PrimeScript^TM^ RT-PCR Kit kit (TAKARA), for miRNA detection, specific miRNA reverse primers and U6 reverse primer were used to reverse transcript mature miRNAs and U6 snRNA, respectively. For pri-miRNA and pre-miRNA detection, random primer was used to reverse transcription. SYBR^®^ Green PCR Master Mix (Applied Biosystems) was used for qPCR on StepOnePlus Real-Time PCR System (Applied Biosystems) to analyze the miRNAs. U6 snRNA was used for normalization of mature miRNA, GAPDH was used for normalization of pri-miRNA and pre-miRNA.

### RNA immunoprecipitation assay (RIP)

The methods were modified from other lab and our lab [52, 53]. Briefly, 293 T stable cell lines were transfected with indicated plasmids for 48 h, and then lysed in the RIPA buffer (50 mM Tris-HCl pH7.4, 150 mM NaCl, 10 mM EDTA, 5 mM MgCl, 1% NP-40, 1 mM DTT, 100 units/ml RNase inhibitor (Fermentas), 400 μM VRC (New England BioLabs) and protease inhibitor cocktail)) for 1 h on ice. Then 1/10 of lysates were extracted with 1 ml of TRIZOL reagent (Invitrogen) for total RNAs as an Input, 1/50 of lysates was saved for Western blotting to detect the protein expression, and the other lysates were incubated with 40 μl of protein A/G-agarose beads and with 4 μl of related antibodies at 4 °C overnight. Beads bound with RNAs were washed with the same RIP-lysis buffer for five times, then 1/10 of beads was subjected to Western blotting analysis to identify the efficiency of IP, the remained beads were extracted with 1 ml of TRIZOL for RIP RNAs. The mature RNAs and pre-miRNAs bound by AGO2 were detected by Northern blotting.

### In situ hybridization (ISH) for microRNA

The miR-19b ISH was conducted as described previously [54, 55]. Briefly, the lung cancer tissue arrays were purchased from ALenabio (Cat#PR956, Xi’an, China), which contained 208 individual clinical lung cancer samples (normal 40, cancer 168). The tissue microarray slide was hybridized with digoxigenin double-labeled LNA (locked nucleic acid)-modified oligonucleotid probe for human hsa-miR-19b (40 μM) for 1 h at 37 °C and then at 4 °C overnight. The following steps included: the slide was stained with anti-DIG antibody in blocking solution for 30 min at 37 °C, and then incubated with the alkaline phosphatase (AP) substrate NBT-BCIP for visualization; the slide was stained with Nuclear Fast Red. All images were captured and processed using identical settings.

### Immunohistochemical staining (IHC)

IHC was performed according to the method as described previously [54, 55]. Briefly, human lung cancer tissue array slides were stained with antibodies against AGO2-K493-Ac and AGO2-K720-Ac at 4 °C overnight, and then incubated with secondary antibody conjugated with HRP at 37 °C for 30 min. The DAB Chromogen Kit was used for visualization of peroxidase catalyzed product (brown), followed by with hematoxylin counterstaining (blue), lastly dehydrated with ethanol and xylene to prepare for mounting. All images were captured and processed using identical settings.

### ISH and IHC scoring

MiR-19b, AGO2-K493-Ac and AGO2-K720-Ac staining results were scored semiquantitatively based on staining intensity and distribution by using the immunoreactive score (IRS) method [56]. The staining intensity (SI) categorized as 0 (negative), 1 (weak), 2 (intermediate) or 3 (strong) and the percentage of positive cells (PP) scored as 0 (0% positive), 1 (1–25%), 2 (26–50%), 3 (51–75%) or 4 (76–100%). The scores were generated by multiplying the staining intensity (SI) and proportion positivity cells (PP).

### Soft agar colony-forming assay

Cellular transformation and tumorigenesis were assessed by a soft agar colony formation assay as previously described [52, 57]. Briefly, six-well plates were added 2 ml of DMEM medium with 10% FBS and with 0.6% agar gel (Amresco) as the base gel. Furthermore, 3 × 10^3^ of each of A549 stable cell lines per well were seeded in 2 ml of DMEM medium with 10% FBS and with 0.35% agar gel onto the layer of base gel as the colony formation gel. Incubate the plates in humidified incubator for 21 days. Photographs were taken after staining with 0.005% crystal violet for 1 h, the colony numbers were counted by Image J.

### Xenograft tumor model

The mouse xenografted tumor model was established as described previously [52, 57]. A 5-week-old male BALB/c nude mice were randomly divided into six groups, each group has five mice. Per nude mouse was injected subcutaneously with 2.5 × 10^6^ of each stable A549 stable cell lines on the bilateral back. Mice were killed on 35 days after infection, and then the tumor were weighted and photographed. All animal studies were conducted with the approval and guidance of Shanghai Jiao Tong University Medical Animal Ethics Committees.

### Statistical analysis

Statistical analyses were performed with Microsoft Excel analysis tools and GraphPad Prism 5. All data are presented as the means ± s.d. for qPCR, soft agar colony assay, mouse xenograft model. Comparisons of the miR-19b, AGO2-K493-Ac and AGO2-K720-Ac levels in subgroups (stages and clinical grades) for significance were analyzed by one way ANOVA. The Spearman correlation analysis was performed to analyze the association between the expressions of miR-19b with AGO2-K493-Ac and AGO2-K720-Ac levels. Comparisons between groups for statistical significance were conducted with a 2-tailed-unpaired Student’s *t*-test. *P* < 0.05(*), *P* < 0.01(**) and *P* < 0.001(***) were considered statistically significant.

## Electronic supplementary material

Dataset 1

Supplemental Figures S1-S8 and Tables S1-S2
