# Supplementary material for: Acetylation of AGO2 promotes cancer progression by increasing oncogenic miR-19b biogenesis
Source: Oncogene. 2018 Oct 10;38(9):1410–31. doi: 10.1038/s41388-018-0530-7 (PMC6372475; doi:10.1038/s41388-018-0530-7)
Supplement: Supplementary file 2 — Supplemental Figures S1-S8 and Tables S1-S2 [file 41388_2018_530_MOESM2_ESM.pdf]

SUPPLEMENTARY FIGURE LEGENDS

Figure S1. AGO2 is Acetylated at K355, K493 and K720, Related to Figure 1

(A) K355, K493 and K720 are identified as the AGO2 acetylation sites by mass spectrometry analysis. 293T cells transfected with Flag-AGO2 were treated with the deacetylase inhibitors TSA (2 μM) and NAM (10 mM) for 6 h and 18 h before harvested, respectively. Flag-AGO2 was purified by IP with anti-Flag antibody, then subjected to 8% SDS-PAGE gel, followed by staining with Coomassie brilliant blue. The band of Flag-AGO2 were cut and digested for the mass spectrometry analysis.

(B-C) Characterization of AGO2 specific acetyl-K355, -K493 and -K720 antibodies. Specificity of AGO2 specific acetyl-antibodies were determined by dot blot assay. Nitrocellulose membrane was spotted with different amounts of acetyl-K355,-K493 and -K720 peptides or unmodified peptides, and detected with antibodies AGO2-K355-Ac, AGO2-K493-Ac and AGO2-K720-Ac, respectively.

(D) AGO2 was acetylated at K355, K493 and K720 in 293T cells. Flag-AGO2 was transfected into 293T cell, AGO2 acetylation analyzed by IP and WB by using AGO2 specific acetylation antibodies with or without the unmodified peptide.

Figure S2. Serum Stimulates the Expression of P300 and Ectopically Expressed P300 Associates with Endogenous AGO2, Related to Figure 2

(A) Serum stimulates AGO2 acetylation. HeLa or A549 cell was serum-starved for 24 h and followed by stimulation with 20% serum for 1, 2, 3 h; the expression of P300 was

measured by WB.

(B) Ectopically expressed P300 associates with endogenous AGO2. 293T cell was transfected with HA-P300, after 48 h cells was lysed with RIPA buffer for IP with anti-HA antibody or normal IgG, followed by WB.

Figure S3. AGO2 Interacts with HDAC7 Rather than Other Deacetylases, Related to Figure 3

Flag-tagged SIRT1, SIRT5, HDAC6 or HDAC7 was individually co-transfected with Myc-AGO2 into 293T cells. Cell lysates were immunoprecipitated with anti-Myc antibody, and followed by WB with anti-Flag antibody (* represents the non-specific band).

Figure S4. Acetylation of AGO2 Increases miR-19b Biogenesis, Related to Figure 4

(A) shRNA-resistant Flag-tagged AGO2-WT and AGO2-3KR were stably re-expressed in A549-shAGO2 cells, respectively. The levels of re-expressed AGO2 were detected by WB and shown comparable.

(B) 293T-shAGO2 and -shDICER stable cell lines. AGO2 and DICER were knocked down by several shRNAs in 293T cells. The protein levels were detected by WB with as indicated antibodies.

(C) The miR-19b maturation is highly sensitive to AGO2 in a DICER dependent manner. DICER or AGO2-knocked down 293T cells were transfected with pre-miR-19b1 or pre-miR-19b2 for 48 h, then total RNAs were extracted and followed by northern blotting analysis.

(D) The miR-19b maturation from pre-miR-19b1 is more sensitive to AGO2 than that from pre-miR-19b2 in a DICER dependent manner. Indicated plasmids were transfected into 293T cells for 48 h, then the total RNA were extracted and followed by northern blotting analysis.

Figure S5. Serum stimulation induces the expression of miR-19b, but not pri-miR-19b1 and pre-miR-19b, Related to Figure 4

A549 cell was serum-starved for 24 h and followed by stimulation with 20% serum for 1, 2, 3 h; the expression of pri-miR-19b1, pre-miR-19b and miR-19b were detected by qRT-PCR (upper panels), and the expression of P300 and AGO2 were measured by WB (lower panels).

Figure S6. Acetylation of AGO2 does not influence its interaction with DICER, Related to Figure 5

AGO2-WT, AGO2-K355R, AGO2-K493R, AGO2-K720R or AGO2-3KR was individually transfected into 293T cells, respectively. 48 h later, cells were lysed with RIPA buffer for IP with anti-Myc antibody, the interaction of endogenous DICER with AGO2 was performed by WB.

Figure S7. AGO2 Acetylation Promotes Caner Progression by miR-19b, Related to Figure 6

(A) Validation of AGO2 expression in A549 stable cell lines. Flag-AGO2-WT, or -3KR

with or without pre-miR-19b1 were stably expressed in A549 cells, and the levels of AGO2 were detected by WB as shown comparable.

(**B**) AGO2 acetylation increases xenograft tumor growth *via* the miR-19b pathway. 2.5x10^6 of above A549 stable cell lines were injected subcutaneously into the back of nude mice. At 35 days tumors were dissected and tumors sizes were shown.

(C) The expression levels of AGO2 in xenograft tumors with A549 stable cell lines. Lysates from xenograft tumors of A549 stable cell lines were determined by WB with anti-AGO2 and anti-Flag antibodies.

Figure S8. AGO2 Acetylation Is Up-regulated and Positively Correlated with miR-19b Expression in Human Lung Cancers, Related to Figure 7

(A) Relevance between the clinical characteristics and the expression levels of miR-19b, AGO2-K493-Ac and AGO2-K720-Ac in lung cancers. Data were analyzed using a one-way ANOVA (within Normal, Stage Ⅰ, Stage Ⅱ and StageⅢ/Ⅳ groups) and independent t-test.

(B-D) The expression levels of miR-19b1, AGO2-K493-Ac and AGO2-K720-Ac in lung cancers were significantly higher than those in normal tissues. ISH staining scores for miR-19b (B) and IHC staining scores for AGO2-K493-Ac (C) and AGO2-K720-Ac (D) in normal tissues and lung cancers were shown. Comparisons between groups for statistical significance were conducted with a 2-tailed-unpaired Student's t-test. The error bars represent mean ±s.d., *P*-values of <0.05 (*), <0.01 (**), <0.001 (***).

Zhang_Fig.S1

A

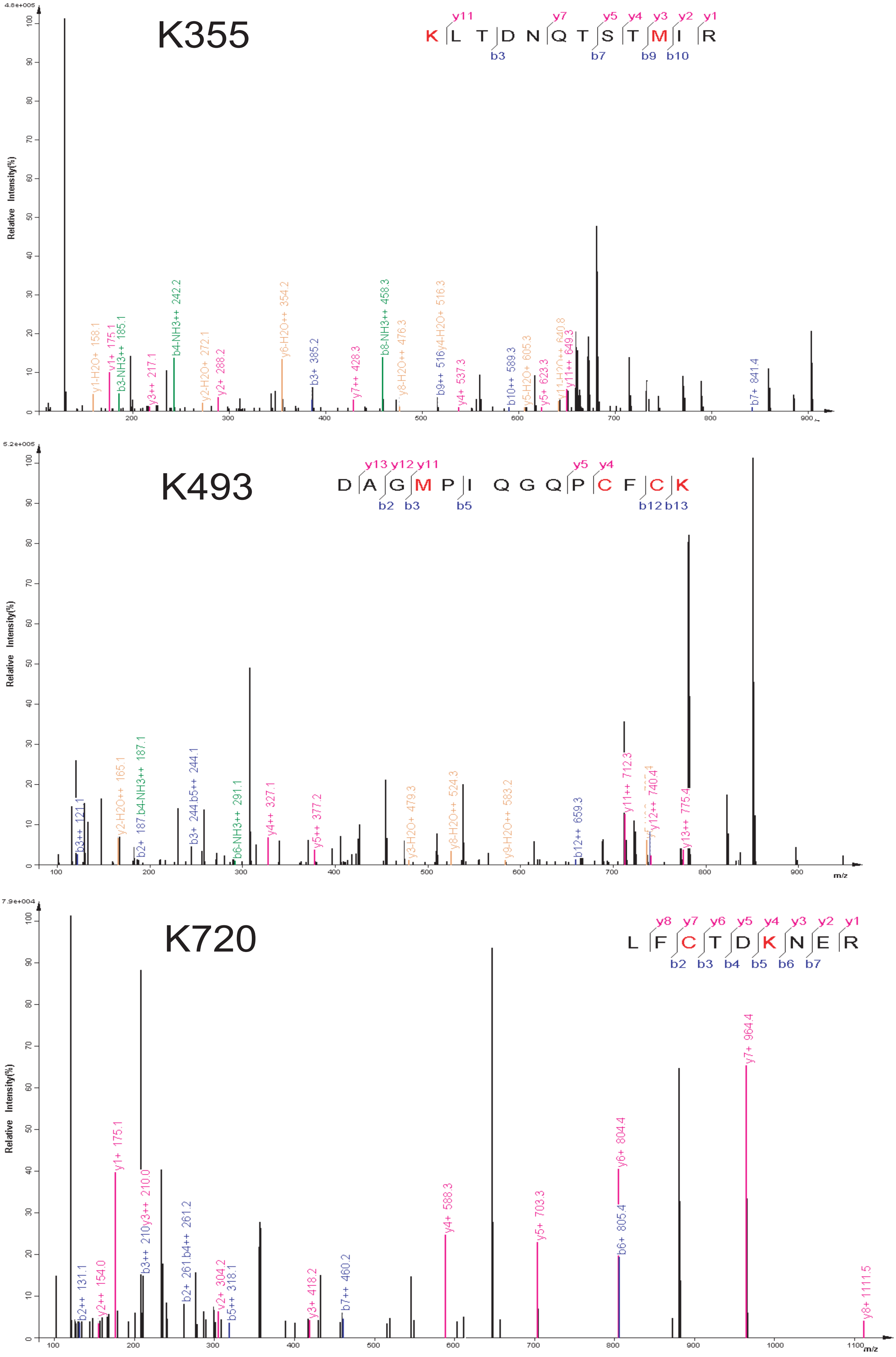

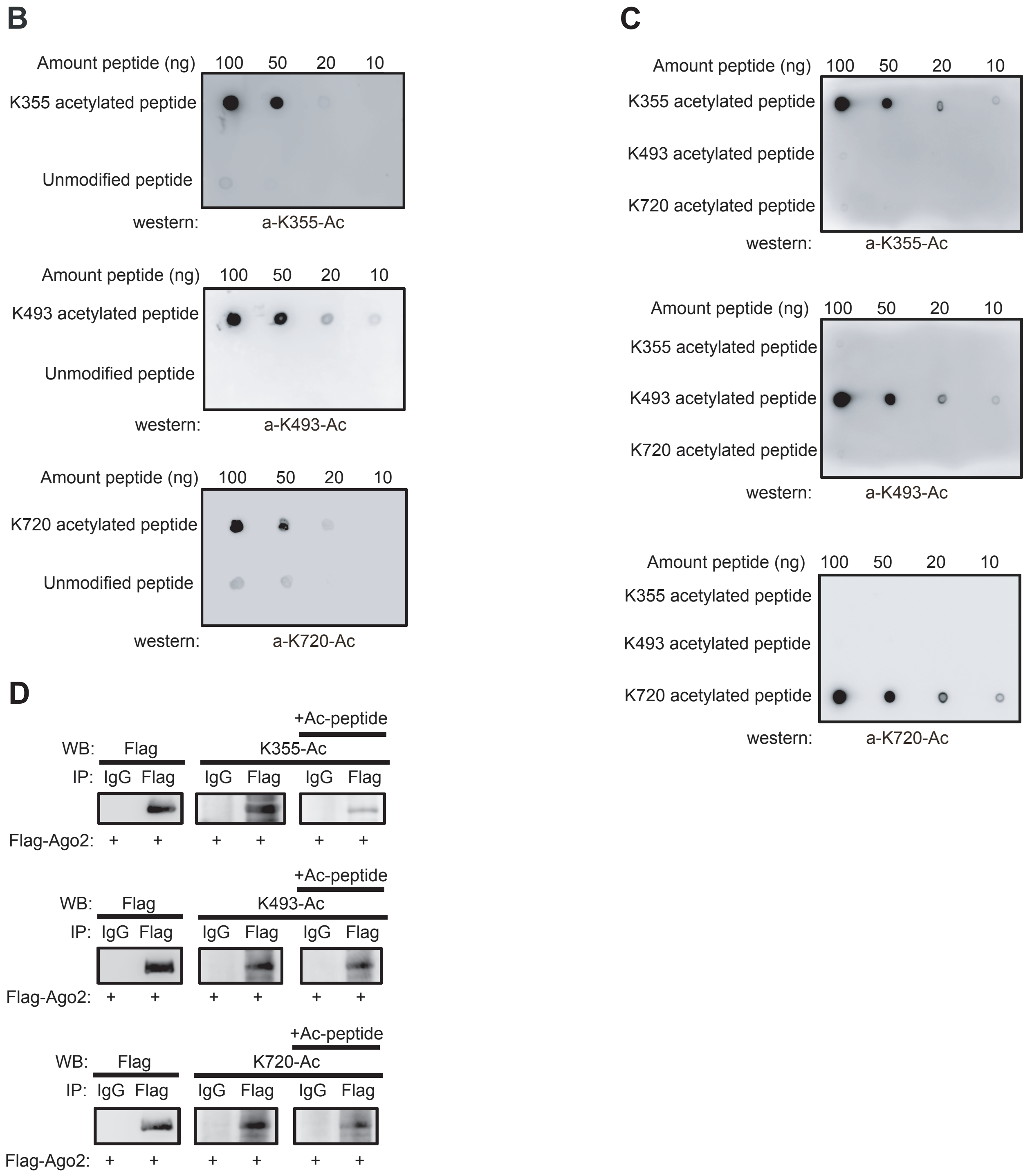
B
Amount peptide (ng) 100 50 20 10
K355 acetylated peptide
Unmodified peptide
western: a-K355-Ac
Amount peptide (ng) 100 50 20 10
K493 acetylated peptide
Unmodified peptide
western: a-K493-Ac
Amount peptide (ng) 100 50 20 10
K720 acetylated peptide
Unmodified peptide
western: a-K720-Ac
C
Amount peptide (ng) 100 50 20 10
K355 acetylated peptide
K493 acetylated peptide
K720 acetylated peptide
western: a-K355-Ac
Amount peptide (ng) 100 50 20 10
K355 acetylated peptide
K493 acetylated peptide
K720 acetylated peptide
western: a-K493-Ac
Amount peptide (ng) 100 50 20 10
K355 acetylated peptide
K493 acetylated peptide
K720 acetylated peptide
western: a-K720-Ac
D
+Ac-peptide
WB: Flag K355-Ac
IP: IgG Flag IgG Flag IgG Flag
Flag-Ago2: + + + + + +
+Ac-peptide
WB: Flag K493-Ac
IP: IgG Flag IgG Flag IgG Flag
Flag-Ago2: + + + + + +
+Ac-peptide
WB: Flag K720-Ac
IP: IgG Flag IgG Flag IgG Flag
Flag-Ago2: + + + + + +

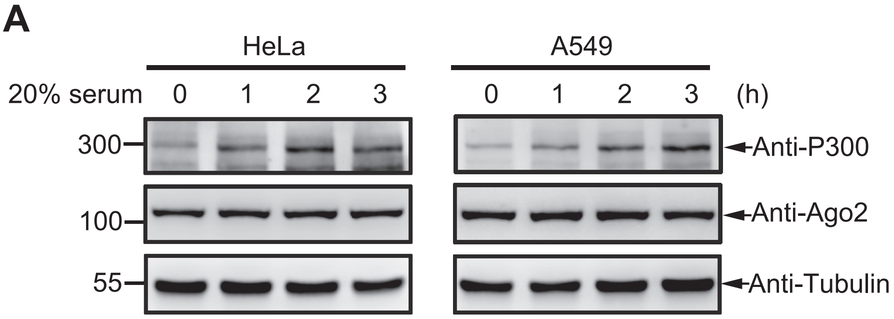
A
HeLa
A549
20% serum
0 1 2 3
0 1 2 3
(h)
300
100
55
Anti-P300
Anti-Ago2
Anti-Tubulin

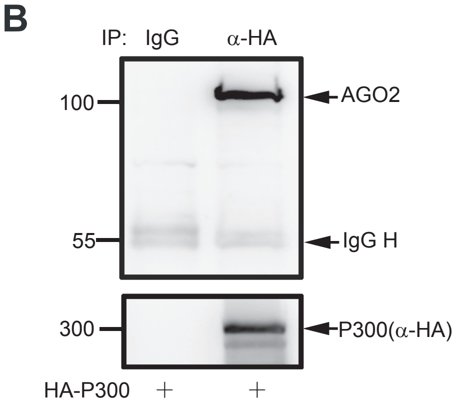
B
IP: IgG α-HA
100
55
300
AGO2
IgG H
P300(α-HA)
HA-P300 + +

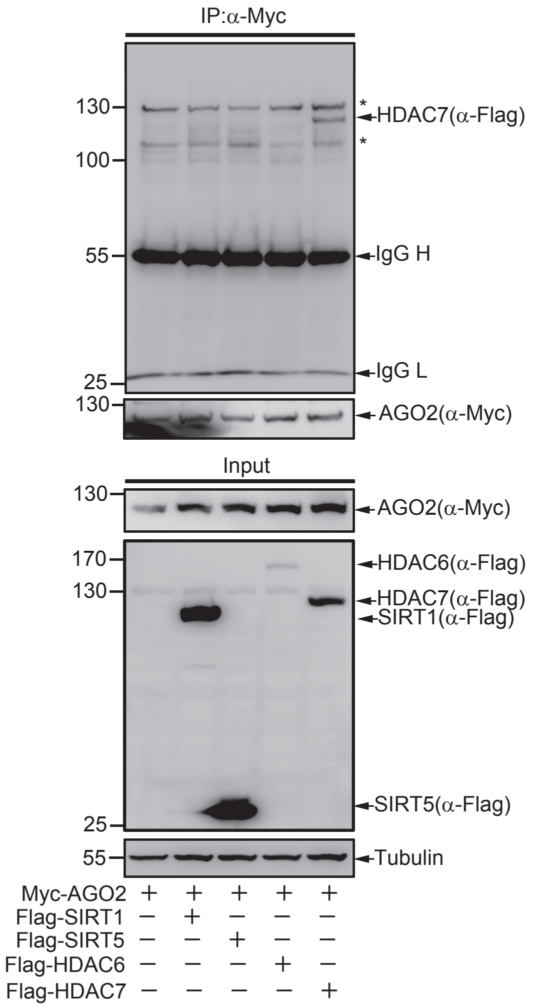
IP:α-Myc
130
100
55
25
*
HDAC7(α-Flag)
*
IgG H
IgG L
130
AGO2(α-Myc)
Input
130
AGO2(α-Myc)
170
130
HDAC6(α-Flag)
HDAC7(α-Flag)
SIRT1(α-Flag)
25
SIRT5(α-Flag)
55
Tubulin
Myc-AGO2 + + + + +
Flag-SIRT1 − + − − −
Flag-SIRT5 − − + − −
Flag-HDAC6 − − − + −
Flag-HDAC7 − − − − +

Zhang_Fig S4

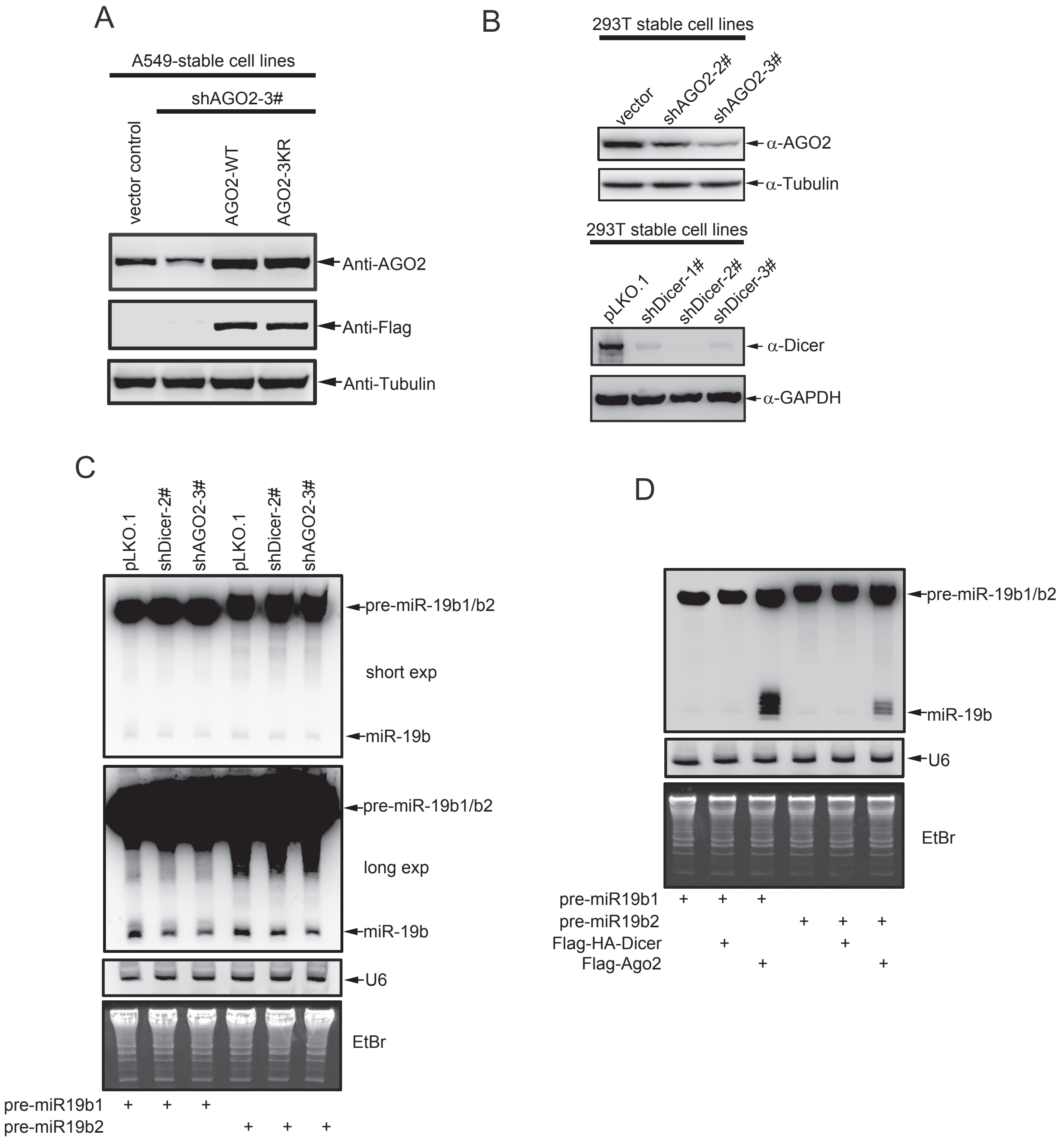
A
A549-stable cell lines
shAGO2-3#
vector control
AGO2-WT
AGO2-3KR
Anti-AGO2
Anti-Flag
Anti-Tubulin
B
293T stable cell lines
vector
shAGO2-2#
shAGO2-3#
α-AGO2
α-Tubulin
293T stable cell lines
pLKO.1
shDicer-1#
shDicer-2#
shDicer-3#
α-Dicer
α-GAPDH
C
pLKO.1
shDicer-2#
shAGO2-3#
pLKO.1
shDicer-2#
shAGO2-3#
pre-miR-19b1/b2
short exp
miR-19b
pre-miR-19b1/b2
long exp
miR-19b
U6
EtBr
pre-miR19b1 + + +
pre-miR19b2 + + +
D
pre-miR-19b1/b2
miR-19b
U6
EtBr
pre-miR19b1 + + +
pre-miR19b2 + + +
Flag-HA-Dicer + +
Flag-Ago2 + +

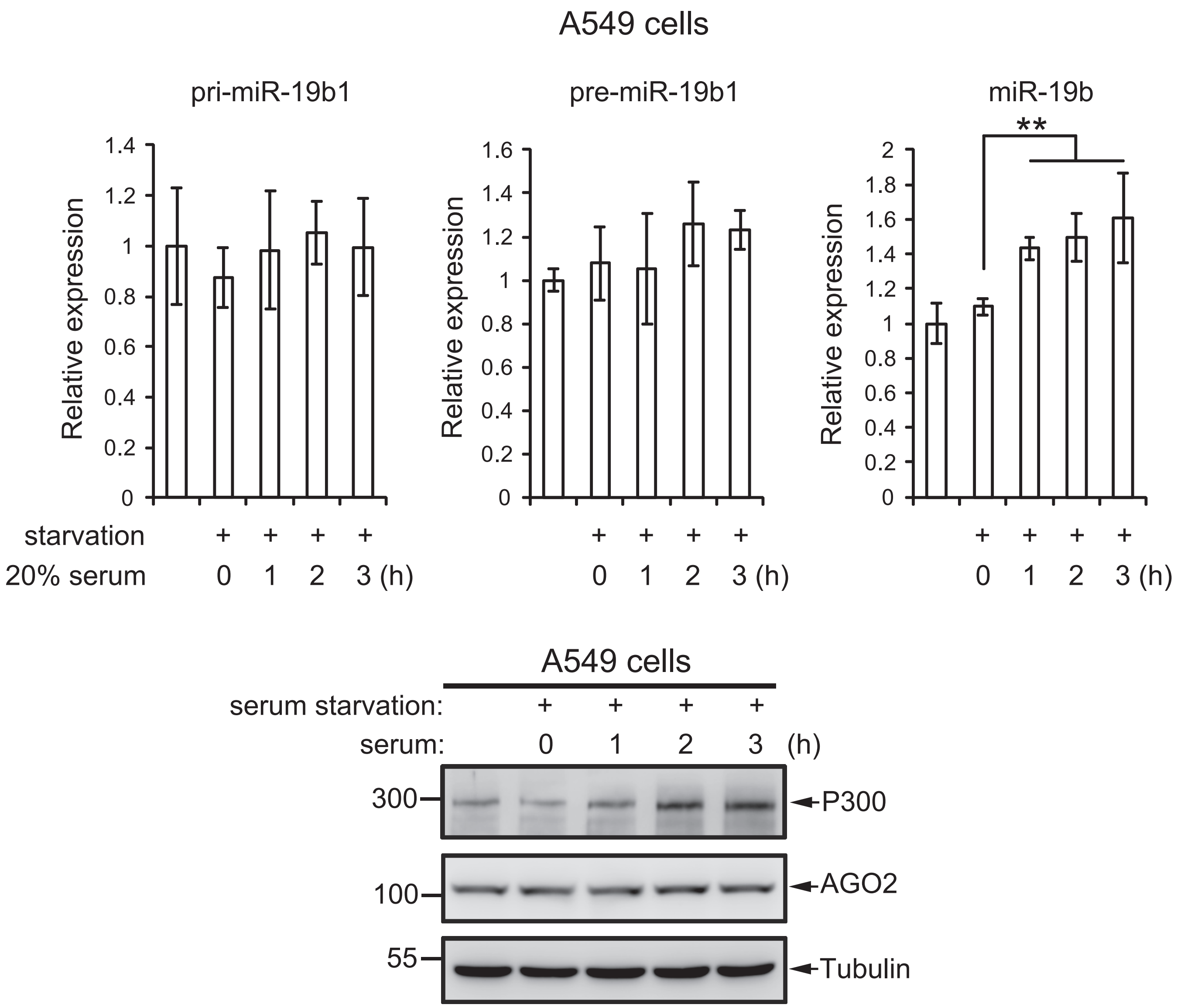
A549 cells
pri-miR-19b1
pre-miR-19b1
miR-19b
Relative expression
**
starvation + + + +
20% serum 0 1 2 3 (h)
A549 cells
serum starvation: + + + +
serum: 0 1 2 3 (h)
300
P300
100
AGO2
55
Tubulin

Zhang_Fig S6

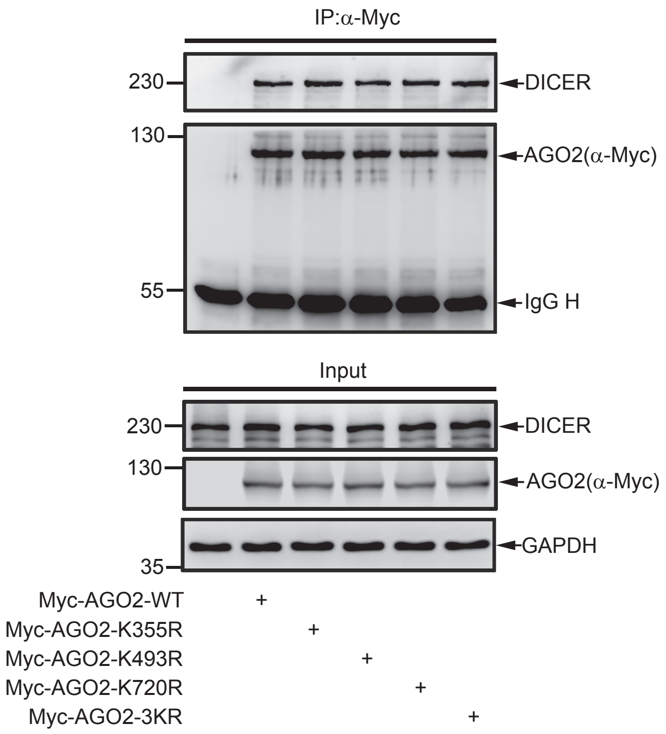
IP:α-Myc
230
DICER
130
AGO2(α-Myc)
55
IgG H
Input
230
DICER
130
AGO2(α-Myc)
GAPDH
35
Myc-AGO2-WT
Myc-AGO2-K355R
Myc-AGO2-K493R
Myc-AGO2-K720R
Myc-AGO2-3KR
+
+
+
+
+

Zhang_Fig. S7

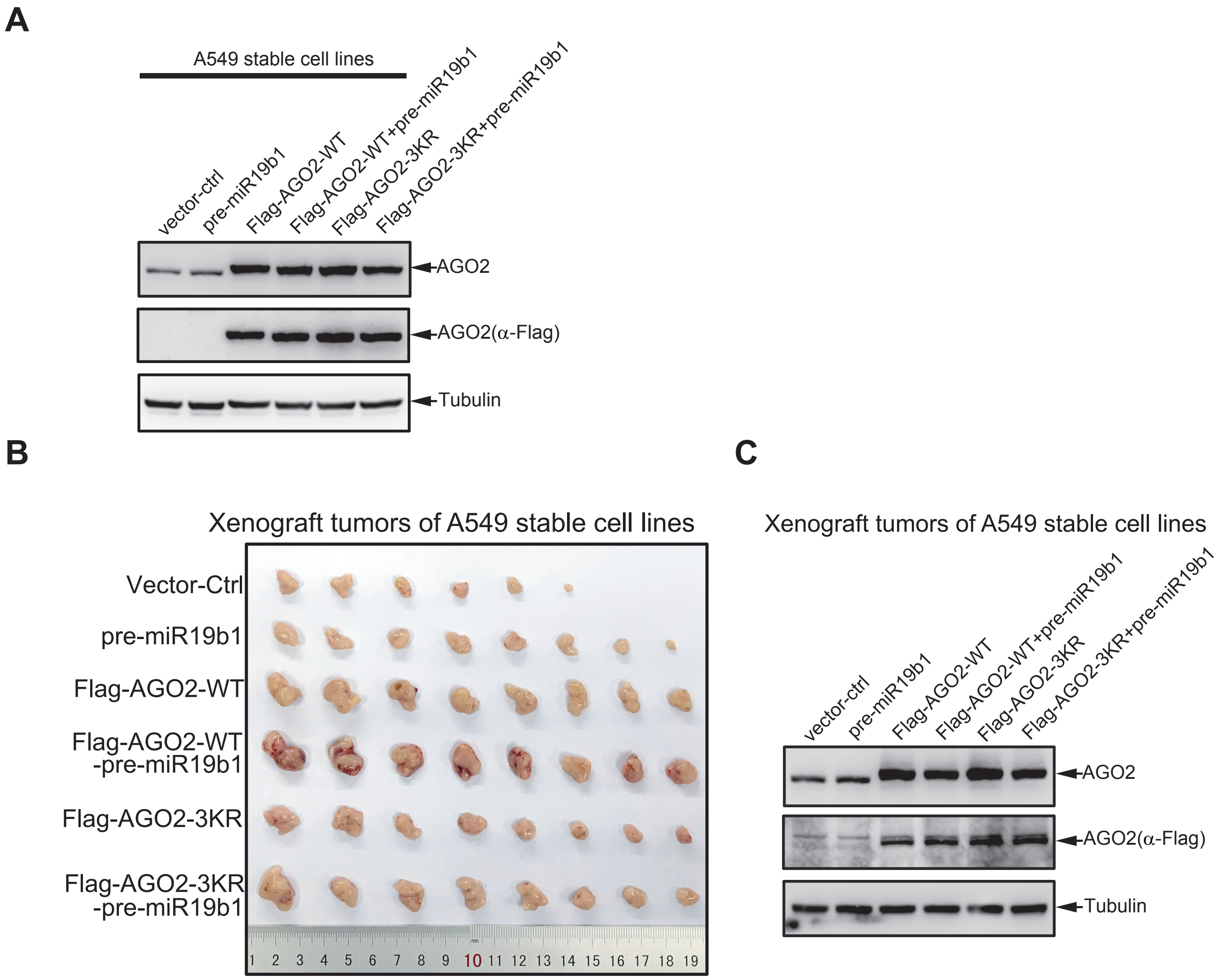
A
A549 stable cell lines
vector-ctrl
pre-miR19b1
Flag-AGO2-WT
Flag-AGO2-WT+pre-miR19b1
Flag-AGO2-3KR
Flag-AGO2-3KR+pre-miR19b1
AGO2
AGO2(α-Flag)
Tubulin
B
Xenograft tumors of A549 stable cell lines
Vector-Ctrl
pre-miR19b1
Flag-AGO2-WT
Flag-AGO2-WT -pre-miR19b1
Flag-AGO2-3KR
Flag-AGO2-3KR -pre-miR19b1
1 2 3 4 5 6 7 8 9 10 11 12 13 14 15 16 17 18 19
C
Xenograft tumors of A549 stable cell lines
vector-ctrl
pre-miR19b1
Flag-AGO2-WT
Flag-AGO2-WT+pre-miR19b1
Flag-AGO2-3KR
Flag-AGO2-3KR+pre-miR19b1
AGO2
AGO2(α-Flag)
Tubulin

A

Relevance between the clinical characteristics and miR-19b, AGO2K493-Ac and AGO2-K720-Ac expression level of lung cancer

	miR-19b			AGO2-K493-Ac			AGO2-K720-Ac		
	N	mean score (95% CI)	P value	N	mean score (95% CI)	P value	N	mean score (95% CI)	P value
Category			< 0.0001			0.0142			0.001
Normal	40	4.3(3.8-4.8)		40	4.8(4.3-5.3)		40	5.3(4.6-5.9)	
Cancer	168	6.4(5.9-6.9)		168	5.9(5.5-6.3)		168	6.6(6.3-7.0)	
Stage			0.0011			0.1082			0.0097
I	85	6.2(5.5-6.9)		85	5.9(5.3-6.5)		85	6.6(6.0-7.2)	
II	44	7.0(6.0-8.1)		44	5.9(5.1-6.8)		44	6.8(6.1-7.6)	
III/IV	39	6.4(5.4-7.4)		39	5.8(5.0-6.6)		39	6.6(6.0-7.3)	
Grade			0.058			0.1662			0.7043
1	38	6.2(5.1-7.2)		38	6.7(5.6-7.7)		38	6.5(5.7-7.4)	
2	66	6.0(5.2-6.7)		66	5.6(5.0-6.3)		66	6.3(5.8-6.8)	
3	33	7.6(6.3-8.8)		33	5.8(4.9-6.7)		33	6.7(5.7-7.7)	

B

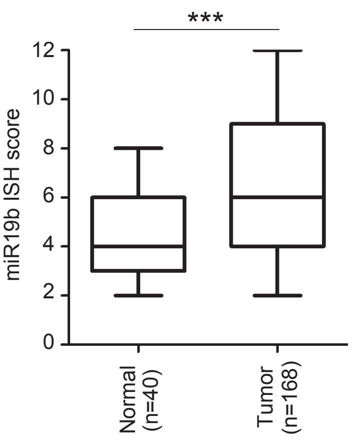

C

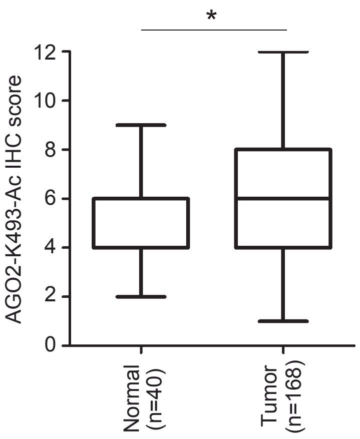

D

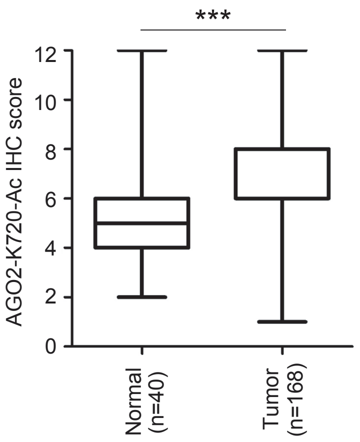

Supplementary Table 1. Related to Figure 1. Alignment of the amino acid sequences of Ago2 homologues in various species.

The conserved lysines of Ago2 were highlighted in red which were identified to be acetylated.
K355, K493 and K720 of Ago2 are evolutionarily conserved from Caenorhabditis elegans to mammals.

Homo sapiens	351RCIKKLTDNQ360	489PCFCKYAQGA498	716FCTDKNERVG725
Pan troglodytes	351RCIKKLTDNQ360	489PCFCKYAQGA498	716FCTDKNERVG725
Mus musculus	352RCIKKLTDNQ361	490PCFCKYAQGA499	717FCTDKNERVG726
Rattus norvegicus	352RCIKKLTDNQ361	490PCFCKYAQGA499	717FCTDKNERVG726
Oryctolagus cuniculus	332RCIKKLTDNQ341	470PCFCKYAQGA479	697FCTDKNERVG706
Bos taurus	352RCIKKLTDNQ361	490PCFCKYAQGA499	717FCTDKNERVG726
Gallus gallus	356RCIKKLTDNQ365	494PCFCKYAQGA503	721FCTDKNERVG730
Sus scrofa	352RCIKKLTDNQ361	490PCFCKYAQGA499	717FCTDKNERVG726
Anolis carolinensis	362RCIKKLTDNQ371	500PCFCKYAQGA509	727FCTDKNERVG736
Macaca mulatta	352RCIKKLTDNQ361	490PCFCKYAQGA499	717FCTDKNERVG726
Felis catus	348RCIKKLTDNQ357	486PCFCKYAQGA495	713FCTDKNERVG722
Desmodus rotundus	352RCIKKLTDNQ361	490PCFCKYAQGA499	717FCTDKNERVG726
Equus caballus	347RCIKKLTDNQ356	485PCFCKYAQGA494	712FCTDKNERVG721
Ailuropoda melanoleuca	350RCIKKLTDNQ359	488PCFCKYAQGA497	715FCTDKNERVG724
Meleagris gallopavo	348RCIKKLTDNQ357	486PCFCKYAQGA495	713FCTDKNERVG722
Ovis aries	346RCIKKLTDNQ355	484PCFCKYAQGA493	712FCTDKNERVG721
Loxodonta africana	345RCIKKLTDNQ354	483PCFCKYAQGA492	710FCTDKNERVG719
Pelodiscus sinensis	352RCIKKLTDNQ361	490PCFCKYAQGA499	718FCTDKNERVG727
Ornithorhynchus anatinus	352RCIKKLTDNQ361	490PCFCKYAQGA499	717FCTDKNERVG726
Bactrocera dorsalis	423RCIKKLTDMQ432	575PCFCKYATGP584	803FCAEKKEQSG 812
Caenorhabditis elegans	515RCIKKL TDVQ524	653PCFCKYAVGV662	880FAVDKKDQVG889

Supplementary Table S2. Primer or oligonucleotide sequences were used in this study

The sequences of siRNA, shRNA, qRT-PCR, probe and plasmid construction		
siRNA		
Negative control	ACGUGACACGUUCGGAGAATT	
si-P300	AGUAAUAUCUUCGUGCCACTT	
si-HDAC7	CCAGCAAACCUUCUACCAATT	
shRNA		
AGO2 shRNA2#	Forword	CCGGTCTATGAACTCAGGGCTTTAAACTCGAGTTTAAAGCCCTGAGTTCATAGTTTTTTG
	Reverse	AATTCAAAAAACTATGAACTCAGGGCTTTAAACTCGAGTTTAAAGCCCTGAGTTCATAGA
AGO2 shRNA3#	Forword	CCGGTATCGAACATGAGACGTCATTGCTCGAGCAATGACGTCTCATGTTCGATTTTTTTG
	Reverse	AATTCAAAAAAATCGAACATGAGACGTCATTGCTCGAGCAATGACGTCTCATGTTCGATA
DICER shRNA1#	Forword	CCGGTGCCAAGGAAATCAGCTAAATTCTCGAGAATTTAGCTGATTTCCTTGGCTTTTTG
	Reverse	AATTCAAAAAGCCAAGGAAATCAGCTAAATTCTCGAGAATTTAGCTGATTTCCTTGGCA
DICER shRNA2#	Forword	CCGGTGGAAGAGGCTGACTATGAAGACTCGAGTCTTCATAGTCAGCCTCTTCCTTTTTG
	Reverse	AATTCAAAAAGGAAGAGGCTGACTATGAAGACTCGAGTCTTCATAGTCAGCCTCTTCCA
DICER shRNA3#	Forword	CCGGTAGCAGCTCTGGATCATAATACCTCGAGGTATTATGATCCAGAGCTGCTTTTTTG
	Reverse	AATTCAAAAAAGCAGCTCTGGATCATAATACCTCGAGGTATTATGATCCAGAGCTGCTA
qRT-PCR primers		
miR-19b	RT	GTCGTATCCAGTGCAGGGTCCGAGGTATTCGCACTGGATACGACTCAGTT
	q-PCR Forword	GCCTGTGTGCAAATCCATGC
	q-PCR Reverse	GTGCAGGGTCCGAGGT
pre-miR-19b	q-PCR Forword	CTATGGTTAGTTTTGCAGGTTTGC

	q-PCR Reverse	CAGTCAGTTTTGCATGGATTTG
pri-miR-19b1	q-PCR Forword	ATCAAACTGTCCTGTTACTG
	q-PCR Reverse	TTCTACAGACTTTTCACTAC
U6	Forword	CGCTTCGGCAGCACATATAC
	Reverse	AGGGGCCATGCTAATCTTCT
GAPDH	Forword	CTCAAGGGCATCCTGGGCTA
	Reverse	ATGAGGTCCACCACCCTGTT
Probe sequences		
miR-19b	TCAGTTTTGCATGGATTTGCACA	
U6	TGTGCTGCCGAAGCGAGCAC	
AGO2 mutant sequences		
AGO2-K355R	Forword	CGATTAACGGACAATCAGACCTC
	Reverse	TTTAATACATCTTTGTCCTGC
AGO2-K493R	Forword	CGATACGCGCAGGGGGCGGACAG
	Reverse	GCAGAAGCACGGCTGGCCCTG
AGO2-K720R	Forword	GAAACGAGCGGGTTGGGAAAAG
	Reverse	TGTCAGTGCAGAAGAGCC
Sequences of pre-miR-19b1 and pre-miR-19b2 constructed in pGreenPuro		
pre-miR-19b1	Forword	GATCCGAGTTTTGCAGGTTTGCATCCAGCTGTGT GATATTCTGCTGTGCAAATCCATGCAAAACTGACT TTTTG
	Reverse	AATTCAAAAAGTCAGTTTTGCATGGATTTGCACA GCAGAATATCACACAGCTGGATGCAAACCTGCAA AACTCG
pre-miR-19b2	Forword	GATCCGAGTTTTGCAGGTTTGCATTTCAGCGTATA TATGTATATGTGGCTGTGCAAATCCATGCAAAACT GACTTTTTG
	Reverse	AATTCAAAAAGTCAGTTTTGCATGGATTTGCACA GCCACATATACATATATACGCTGAAATGCAAACCT GCAAAACTCG
pre-miR-19b1 mutant sequences		
pre-miR-19b1-Δug	Forword	TGTGATATTCTGCTGTGCAAATC
	Reverse	GCTGGATGCAAACCTGCAAAAC
pre-miR-19b1-Δugug	Forword	TGATATTCTGCTGTGCAAATC
	Reverse	GCTGGATGCAAACCTGCAAAAC

pre-miR-19b1-Δugugug	Forword	ATATTCTGCTGTGCAAATCC
	Reverse	GCTGGATGCAAACCTGCAAAAC
pre-miR-19b1-ug*au*ug	Forword	TGATTGATATTCTGCTGTGCAAATCC
	Reverse	GCTGGATGCAAACCTGCAAAAC
pre-miR-19b1-*auau*ug	Forword	ATATTGATATTCTGCTGTGCAAATCC
	Reverse	GCTGGATGCAAACCTGCAAAAC
pre-miR-19b1-*auauau*	Forword	ATATATATATTCTGCTGTGCAAATCC
	Reverse	GCTGGATGCAAACCTGCAAAAC
